# Polydopamine-modified black phosphorus nanosheet drug delivery system for the treatment of ischemic stroke

**DOI:** 10.1093/rb/rbae046

**Published:** 2024-05-02

**Authors:** Shujiang Yin, Jing Hou, Jie Li, Caiyun Zeng, Shuang Chen, Han Zhang, Xing Tian

**Affiliations:** Key Laboratory of Xinjiang Phytomedicine Resource and Utilization, Ministry of Education, College of Pharmacy, Shihezi University, Shihezi 832002, China; Key Laboratory of Xinjiang Phytomedicine Resource and Utilization, Ministry of Education, College of Pharmacy, Shihezi University, Shihezi 832002, China; Key Laboratory of Xinjiang Phytomedicine Resource and Utilization, Ministry of Education, College of Pharmacy, Shihezi University, Shihezi 832002, China; Key Laboratory of Xinjiang Phytomedicine Resource and Utilization, Ministry of Education, College of Pharmacy, Shihezi University, Shihezi 832002, China; Key Laboratory of Xinjiang Phytomedicine Resource and Utilization, Ministry of Education, College of Pharmacy, Shihezi University, Shihezi 832002, China; College of Physics and Optoelectronic Engineering, Shenzhen University, Shenzhen 518060, China; Key Laboratory of Xinjiang Phytomedicine Resource and Utilization, Ministry of Education, College of Pharmacy, Shihezi University, Shihezi 832002, China

**Keywords:** black phosphorus nanosheets, polydopamine, pH response, blood–brain barrier, ischemic stroke

## Abstract

Black phosphorus (BP), as a representative metal-free semiconductor, has been extensively explored. It has a higher drug loading capacity in comparison to conventional materials and also possesses excellent biocompatibility and biodegradability. Furthermore, BP nanosheets can enhance the permeability of the blood–brain barrier (BBB) upon near-infrared (NIR) irradiation, owing to their photothermal effect. However, the inherent instability of BP poses a significant limitation, highlighting the importance of surface modification to enhance its stability. Ischemic stroke (IS) is caused by the occlusion of blood vessels, and its treatment is challenging due to the hindrance caused by the BBB. Therefore, there is an urgent need to identify improved methods for bypassing the BBB for more efficient IS treatment. This research devised a novel drug delivery approach based on pterostilbene (Pte) supported by BP nanosheets, modified with polydopamine (PDA) to form BP-Pte@PDA. This system shows robust stability and traverses the BBB using effective photothermal mechanisms. This enables the release of Pte upon pH and NIR stimuli, offering potential therapeutic advantages for treating IS. In a middle cerebral artery occlusion mouse model, the BP-Pte@PDA delivery system significantly reduced infarct size, and brain water content, improved neurological deficits, reduced the TLR4 inflammatory factor expression, and inhibited cell apoptosis. In summary, the drug delivery system fabricated in this study thus demonstrated good stability, therapeutic efficacy, and biocompatibility, rendering it suitable for clinical application.

## Graphical Abstract

**Figure:**
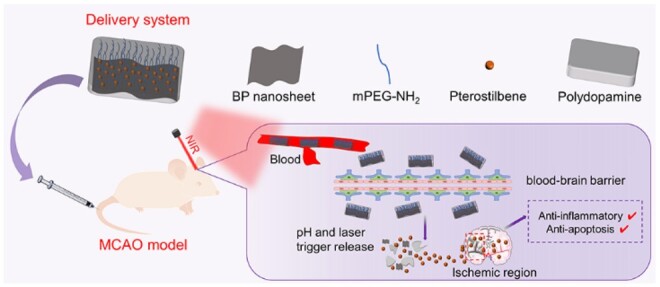

## Introduction

Stroke manifests as a neurological deficit due to sudden vascular or blood irregularities, disrupting blood circulation in the brain. It is characterized by a high prevalence, recurrence, disability, and mortality rates. Stroke is one of the leading causes of death and long-term disability in the world [1], claiming 1.57 million deaths in 2018, which constituted 22.33% of the total mortality [2]. Clinically, strokes are categorized into Ischemic stroke (IS) and hemorrhagic stroke (HS), with IS accounting for approximately 70% and being the primary stroke type [3]. IS occurs due to the abrupt blockage of blood vessels, leading to inadequate oxygenated blood flow and a dearth of nutrients reaching the brain [4]. Despite a survival rate exceeding 50%, stroke remains a predominant cause of physical impairment [5], contributing to nervous system dysfunctions and compromising both physical capabilities and cognitive functions, such as learning, memory, and overall cognition. The clinical treatment strategies for stroke mainly include early intravenous thrombolysis, mechanical thromboplasty, neuroprotection, and neurorestoration [6–8]. However, the cumulative efficiency of drugs is low and the targeting is poor, with most drugs failing to achieve significant efficacy because they cannot effectively pass through the blood–brain barrier (BBB) [9, 10].

Facilitating selective drug transport across the BBB remains a significant challenge in enhancing drug efficacy. Nanocarriers have emerged as a principal strategy to overcome these hurdles [11–13]. However, conventional nanocarriers still face limitations in effectively combatting IS. Major issues include the inadequate targeting of liposomes, the high cost of preparation, potential toxicity associated with polymer nanoparticles [14, 15], and the poor stability of polymeric micelles. Among the emerging two-dimensional materials, the honeycomb folding structure of black phosphorus (BP) can effectively increase the drug-loading capacity of drugs and biomolecules [16–18]. This material is easily degradable in physiological environments, posing no toxicity concerns for the body. Its superior biodegradability and biocompatibility give it a competitive edge over other two-dimensional materials. Research demonstrates BP’s efficacy in photothermal and photodynamic therapy upon near-infrared (NIR) irradiation [19]. Additionally, BP nanosheets exhibit the ability to increase BBB permeability through photothermal effects following NIR exposure. The inherent instability of BP can, however, compromise its characteristics limiting its potential applications. To address this, various methods can enhance its stability and preserve its attributes [20]. For instance, coating the BP surface can prevent air oxidation, thereby preserving its integrity and characteristics [21]. Li *et al.* [22] successfully prepared a bionic peptide-modified BP nanosheet inspired by mussels, which substantially improved the stability of BP. Research indicates that the mussel-inspired polydopamine (PDA) effectively boosts the stability of BP nanosheets while also enabling surface modifications for targeted molecules and long-term circulating agents *in vivo* [23]. PDA exhibits pH responsiveness, maintaining stability under neutral pH conditions but degrading in more acidic microenvironments, thereby facilitating drug release [24]. Therefore, the modification of PDA on the surface of BP nanosheets can not only improve the stability of BP but also transform it into an ideal platform for delivering therapeutic drugs to patients with IS.

Pterostilbene (Pte) (3,5-dimethoxy-4′-hydroxy stilbene) is a natural dimethylated analogue of resveratrol (3,5,4′-trihydroxy stilbene), found primarily in blueberries and grapes [25]. Pte has low molecular weight and robust lipophilicity, facilitating its easier traversal across the BBB and ensuring enhanced bioavailability [26]. With regards to safety, Pte exhibits minimal toxic side effects and is categorized as low risk. *In vivo* studies have revealed Pte’s antioxidant, anti-apoptotic, anticancer, and anti-inflammatory properties [27], making Pte a potential therapeutic agent for treating various diseases, including IS. Badruddeen *et al.* [28] found that Pte affected reperfuse-induced ischemia and related behavioral changes in rats. Owing to its antioxidant and free radical scavenging properties, and its ability to regulate neurodegenerative brain oxidative damage, Pte can be used as a neuroprotective adjuvant to effectively eradicate oxidative stress during *in vivo* ischemic injury [29, 30]. Overall, it IS reasonable to consider Pte as a potential candidate therapeutic agent to prevent or even treat IS.

**Figure 1.**
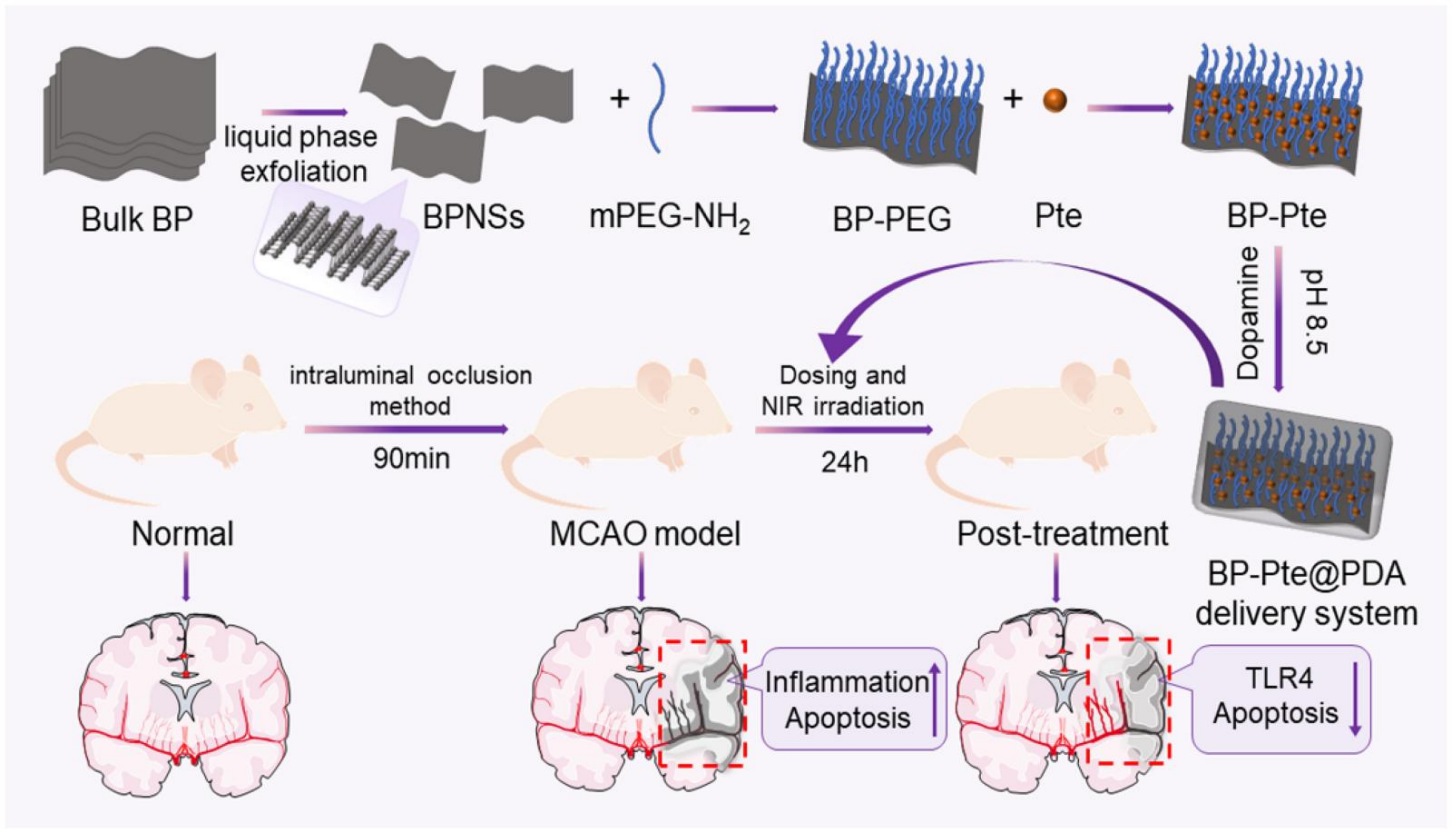
Preparation of BP-Pte@PDA delivery system and its schematic diagram for treatment of IS.

This study designed a PDA-modified BP drug-loaded nano-drug delivery system BP-Pte@PDA for the treatment of IS (Figure 1). Pte demonstrates anti-inflammatory, anti-apoptotic, and neuroprotective properties. The surface modification of BP nanosheets using PDA enhances BP stability and serves as a pH-controlled release mechanism, degrading specifically in ischemic brain regions to release drugs, effectively combating IS. Moreover, *in vivo*, NIR irradiation increases BBB permeability within the drug delivery system. Altogether, this approach offers a safe, dependable preparation capable of crossing the BBB, presenting a novel avenue for treating IS clinically.

## Materials and methods

### Materials

BP powder was purchased from Nanjing Xianfeng Nanomaterial Technology Co., LTD. *N*-methylpyrrolidone (NMP) was purchased from Shanghai Maclin Biochemical Technology Co., LTD. mPEG-NH_2_ was purchased from Shanghai Yiyi Biotechnology Co., LTD. Pte and dopamine hydrochloride were procured from Shanghai McLean Biochemical Technology Co., LTD. Phosphate buffer solution (PBS) was purchased from Shenggong Bioengineering (Shanghai) Co., LTD. CY5.5-PEG-NH_2_ was purchased from Guangzhou Carbonwater Technology Co., LTD. Saline was purchased from Sichuan Kelun Pharmaceutical Co., LTD. Triphenyltetrazolium chloride (TTC) was acquired from Beijing Solaibao Technology Co., LTD. Beijing Langeco Technology Co., LTD supplied 4% paraformaldehyde. DMEM, CCK8 assay kit, and H&E staining kit were respectively purchased from Gibco, Invigentech, and Beijing Solaibao Technology Co., LTD.

### Preparation of BP nanosheets

BP nanosheets were prepared by liquid-phase exfoliation. The BP powder underwent comprehensive grinding in a mortar, followed by treatment at a consistent temperature of 10°C for 48 h using an ultrasonic cleaner (300 W) assisted by a circulating water cooler. Subsequently, centrifugation at 5500 rpm/min for 25 min was carried out. The supernatant was collected, centrifuged at 15 000 rpm/min for 25 min, precipitated, and 1 ml NMP was added to obtain BP nanosheet solution.

### Preparation of BP nanosheets loaded with Pte (BP-Pte)

BP nanosheets with a mass ratio of 1:10 were mixed with mPEG-NH_2_ and stirred continuously at room temperature for 12 h in a light-protected environment. The sample was centrifuged at 12 000 rpm/min at 4°C for 20 min after which supernatant was removed and the precipitate was collected. BP nanosheets with a mass ratio of 1:6 were mixed with Pte, and the resulting precipitate and Pte solution were mixed continuously at room temperature for 24 h in the dark. The samples were centrifuged at 12 000 rpm/min at 4°C, for 25 min. Using the same centrifugal conditions, the sample was cleaned twice with PBS. The precipitate was gathered and the product was named BP-Pte.

Determination of drug loading ratio: The BP nanosheets and Pte were mixed in mass ratios of 1:2, 1:4, 1:6, 1:8 and 1:10, and the solution was thoroughly mixed. After stirring in the dark for 24 h, the mixture was centrifuged at 12 000 rpm/min, 4°C, for 25 min to remove the precipitate. The supernatant, obtained after centrifugation and washing, was collected, and the Pte concentration was measured using an ultraviolet spectrophotometer, using the absorption standard curve. The UV absorption wavelength was set at 307.4 nm. Formula (1) for calculating the drug loading rate is as follows:

(1) $$\mathrm{Loading}\;\mathrm{ratio}\left(\%\right)=\frac{m-m_{1}}{m}\;\times \;100\%\;$$

where *m* and *m*_1_ respectively represent the amount of the original Pte, the amount of Pte in the supernatant and the washing solution.

### Preparation of PDA-modified BP-Pte (BP-Pte@PDA)

Dopamine hydrochloride 7.5 times the mass of BP nanosheets was introduced into the BP-Pte solution, and the pH was set to 8.5. The samples were stirred at room temperature for 12 h away from light. Afterward, the samples underwent centrifugation at 12 000 rpm/min, 20°C, for 25 min, and the supernatant was discarded. Following the same centrifugation parameters, the sample was rinsed twice with PBS. The resulting product derived from the precipitate was labeled BP-Pte@PDA.

### Characterization of BP preparations

BP, BP-Pte and BP-Pte@PDA were characterized by transmission electron microscopy (TEM), scanning electron microscopy (SEM), high-resolution scanning TEM, Fourier transform infrared spectroscopy (FTIR), ultraviolet-visible spectrophotometry (UV-vis) and Malvern laser particle size analyzer.

BP, BP-Pte and BP-Pte@PDA were ultrasonically dispersed in PBS, then dripped onto a copper net and dried at room temperature for 12 h. A TEM (Hitachi HT7700) with an accelerated voltage of 80 kV was used to detect the micromorphology of the samples. The BP-Pte@PDA drug delivery system was simultaneously irradiated with NIR in a solution with pH = 5, degraded for 48 h, and characterized by TEM. The solution droplets were applied onto the silicon wafer, and the microstructure was examined using a SEM (Hitachi SU8010) operating at an accelerated voltage of 15 kV. For detailed observation of the morphology and elemental analysis, high-resolution scanning TEM (FEI Tecnai G2 F20) was employed. FTIR analysis (Bruker Vertex 70v) was conducted through the KBr disc method across the 4000–500 cm^−1^ range to obtain the infrared spectrum of the sample. UV-vis measurements (UV-2600 model) captured the full wavelength variation from 200 to 500 nm. Additionally, the particle size, polymer dispersity index (PDI) and Zeta potential value were determined at 25°C using a Malvern laser particle size analyzer.

### Photothermal performance evaluation

PBS, BP and BP-Pte@PDA solutions (100 μg/ml) were irradiated with NIR (808 nm, 1.0 W/cm^2^) for 10 min, and the temperature was recorded every 10 s, and the temperature changes of each solution were compared. BP and BP-Pte@PDA solutions (100 μg/ml) were irradiated with NIR (808 nm) at different powers (0.5, 1.0, 1.5 and 2.0 W/cm^2^) for 10 min. The temperature was recorded every 10 s, and the temperature changes of each solution were compared. To evaluate the effect of concentration on photothermal performance, BP and BP-Pte@PDA solutions of different concentrations (50, 100, 150, 200 μg/ml) were irradiated with NIR (808 nm, 1.0 W/cm^2^) for 10 min. The temperature was recorded every 10 s, and the temperature changes of each solution were compared. BP and BP-Pte@PDA solutions (100 μg/ml) were exposed to NIR irradiation (808 nm, 1.0 W/cm^2^) for 10 min, followed by natural cooling, constituting a single cycle. Five cycles of this process were performed in total, recording the temperature every 10 s and comparing the peak temperature changes for each cycle of the solutions.

### Stability evaluation

Appropriate amounts of BP, BP-Pte and BP-Pte@PDA solutions were placed at room temperature away from light, and full-wavelength UV scanning at 200–500 nm was performed at 1, 4 and 7 days, respectively. Zeta potential values were also measured. Stability changes were evaluated by comparing absorption curves and changes in Zeta potential values. The BP and BP-Pte@PDA solutions underwent 808 nm NIR irradiation (power density: 1 W/cm^2^) for 10 min, followed by natural cooling, constituting one cycle. This cycle was repeated five times. Ultraviolet full wavelength scanning at 200–500 nm was conducted before and after the light exposure cycles to compare the absorption curves and assess the change in photothermal stability.

### Study on drug release *in vitro*

BP-Pte@PDA (2 mg) was placed in a dialysis bag and incubated in 50 ml 30% ethanol solution at different pH (pH = 5, 6, 7). At pre-determined time points of 0, 0.167, 0.5, 1, 2, 4, 6, 8, 12, 24, 36 and 48 h, a 2-ml volume of external solution was collected, and the absorbance at 307.4 nm was detected to quantify the release of Pte. The Pte release of BP-Pte@PDA solution irradiated with NIR was measured using the same method however before dialysis, the samples were exposed to 808 nm NIR irradiated for 10 min (power density: 1 W/cm^2^). The formula (2) for calculating the drug release rate *in vitro* is as follows:

(2) $$\mathrm{Drug}\;\mathrm{release}\left(\%\right)=\frac{\mathrm{Drug}\;\mathrm{release}\;\mathrm{amount}}{\mathrm{Total}\;\mathrm{drug}\;\mathrm{amount}}\;\times \;100\%\;$$

### Animal experiment

SPF-grade male Kunming mice, aged 6–8 weeks and weighing 25 ± 5 g, were procured from Henan Skibes Biotechnology Co., LTD., and acclimated in an appropriate environment for 1 week with *ad libitum* access to food and water. The animal protection guidelines used in this experiment were formulated by the Animal Protection Committee of the First Affiliated Hospital of Shihezi University, and the animal welfare and experimental process were conducted following the provisions of the Animal Ethics Committee of the First Affiliated Hospital of Shihezi University (No. A2023-252-01).

### Hemolysis test

Mouse blood was centrifuged at 1500 rpm for 10 min to isolate the red blood cells (RBCs). Separately, an appropriate volume of normal saline underwent centrifugation at 2000 rpm/min for 5 min, followed by 3–4 washes until the supernatant became colorless and clear. The purified mouse RBCs were suspended at a 2% concentration (v/v). A 0.1-ml portion of the 2% RBCs suspension was mixed with 0.1 ml each of pure water, normal saline, and varied concentrations (50, 100, 150, 200 μg/ml) of BP, BP-Pte and BP-Pte@PDA solutions. Then, the samples were incubated at 37°C for 3 h, centrifuged at 2500 rpm for 5 min, and the supernatant was separated. The absorbance at 540 nm was detected with an enzyme marker, and the hemolysis rate was calculated according to formula (3):

(3) $$\text{Hemolysis}（\%）=\frac{A_{t}-A_{\text{nc}}}{A_{\text{pc}}-A_{\text{nc}}}\;\times \;100\%\;$$

where *A*_t_ is the absorbance of the solution to be tested, *A*_nc_ is the absorbance of the negative control and *A*_pc_ is the absorbance of the positive control. If the observed hemolysis rate exceeds 5%, it indicates the presence of hemolysis.

### Construction of a mouse model of ischemic stroke

Most researchers have validated estrogen’s neuroprotective effects against cerebral ischemic injury and have observed smaller cerebral infarction sizes of female mice compared to age-matched males in the middle cerebral artery occlusion (MCAO) model [31–33]. Consequently, to preserve the integrity of experimental data, male mice were chosen as the model animals. The MCAO mouse model was induced using the thread peg method. Before the procedure, mice were weighed, anesthetized with intraperitoneal injections of 0.1 ml/10 g 0.3% pentobarbital solution, and securely positioned in a supine orientation on the operating table. Neck hair were removed, and the neck was disinfected with 75% alcohol. A midline incision was made in the neck to access and separate the common carotid artery (CCA), external carotid artery (ECA), and internal carotid artery (ICA), ensuring that the vagus nerve, glossopharyngeal nerve, and trachea remain safe from any injury. A nylon suture was used to ligate the proximal end of the CCA, about 8 mm from the point, where the CCA forks. The distal end of the ECA was then lapped and the distal end of the ICA was temporarily clamped using an arteriolar clamp. A suture was pre-placed at the CCA trunk, approximately 1 mm away from the CCA fork, and a small incision was cut at the CCA trunk about 4 mm away from the CCA fork, using microsurgical scissors. The thread plug was carefully inserted into the CCA along the incision with microforceps, and it was secured in place with a pre-positioned knot to prevent it from slipping out. Subsequently, the arteriolar clamp on the ICA was loosened, and the thread plug was slowly advanced, stopping if resistance was encountered. Once the procedure was completed, the cervical submaxillary gland was repositioned, and the neck skin was sutured. Additionally, the stump of the thread plug was marked with a black marker to aid in its identification during the removed process after reperfusion. After 90 min of cerebral ischemia, the cord plug was gently pulled out and stopped when it met resistance. At this point, the head end of the cord plug reached the incision in the CCA, and any excess cord plug protruding from the skin was trimmed off. However, the cord was not entirely removed to prevent potential bleeding arising from CCA defects. In the sham group, only the operation was performed and no thread plugs were inserted [34].

The animal’s anal temperature was maintained at about 37°C during the entire procedure. The procedure typically takes around 10–15 min. If it exceeds 15 min, involves excessive bleeding, or if the anesthesia depth becomes too intense, the animals are deemed unsuitable and should be abandoned and excluded from the study. After the operation, the mice were placed in a feeding cage with clean bedding. The room temperature was maintained at 25 ± 2°C, and they were allowed free access to food and water. The success of the model was indicated by the presence of the Horner sign in the right eye. In addition, the inability to straighten the left forelimb adduction when lifting the tail, as well as tipping to the left or turning counterclockwise while crawling, were observed to eliminate deceased or unsuccessful individuals. The entire scoring process was conducted under single-blind conditions, meaning that the raters were unaware of the grouping of experimental animals.

### Fluorescence imaging

BP@PDA and CY5.5-PEG-NH_2_ solution were mixed in a mass ratio of 1:10, stirred at room temperature away from light for 12 h, and then centrifuged at 12 000 rpm/min for 25 min to remove the supernatant, and washed with PBS for two or three times. The precipitate is taken and the resulting product is referred to as BP@PDA-CY5.5.

The mice were randomly divided into three groups (*n* = 4): (i) Model+CY5.5+NIR irradiation; (ii) Model+BP@PDA-CY5.5; (iii) Model + BP@PDA-CY5.5+NIR irradiation. Following the successful establishment of the mouse MCAO model, the designated solutions (CY5.5 1 mg/kg, BP 6 mg/kg) were administered. At 30 min, all animals except those in group 2 underwent NIR irradiation (808 nm, 1 W/cm^2^) for 10 min. Subsequent fluorescence imaging was conducted on mice at 2, 4, 6, 8, 12 and 24 h post-injection. Additionally, a small subset of animals (*n* = 3) were anesthetized every 6 h, and major organs (heart, liver, spleen, lung, kidney and brain) were isolated via normal saline perfusion for further analysis. After washing, fluorescence imaging was performed, and fluorescence intensity was quantified and compared using Image J software.

### Pharmacokinetic analysis

The mice were randomly divided into two groups (*n* = 3): (i) Model +Pte; and (ii) Model + BP-Pte@PDA+ NIR irradiation. After the successful establishment of the MCAO model in mice, two groups of mice were administered Pte and BP-Pte@PDA solutions via the tail vein at a dosage of 14 mg/kg Pte, respectively. Subsequently, both groups underwent NIR irradiation (808 nm, 1 W/cm^2^) for 10 min. Plasma samples were collected at intervals of 0.083, 0.25, 0.5, 1, 2, 4, 6, 8, 12 and 24 h post-administration, while brain tissue samples were obtained at 0.25, 0.5, 1, 2, 4, 6, 8, 12 and 24 h post-cardiac perfusion. Pte concentrations in all samples were measured by UPLC. In addition, DAS (Drug and Statistics) software was used to calculate pharmacokinetic parameters, including peak Pte concentration (*C*_max_), elimination half-life (*T*_1/2_), area under the concentration-time curve (AUC_0-__*t*_), and mean retention time (MRT_0-__*t*_) in plasma and brain tissue.

### Cerebral infarction evaluation

The mice were randomly divided into five groups (*n* = 3): (i) Control; (ii) Model; (iii) Model + Pte; (iv) Model + BP-Pte@PDA; (v) Model + BP-Pte@PDA+NIR irradiation. Following the successful establishment of the mouse MCAO model, mice in groups 3–5 received an injection via the tail vein containing the specified solutions at a dose of 14 mg/kg Pte. After 30 min, mice in group 5 underwent NIR irradiation (808 nm, 1 W/cm^2^) for 10 min. 24 h later, following cardiac perfusion, brain tissue samples were collected, washed, frozen for 2 h, sliced, and stained with TTC. The infarct rate was calculated according to formula (4):

(4) $$\mathrm{Infarct}\;\mathrm{volume}\left(\%\right)=\frac{\mathrm{Infarction}\;\mathrm{area}}{\mathrm{Total}\;\mathrm{area}}\;\times \;100\%\;$$

### Brain water content evaluation

The mice were randomly divided into five groups (*n* = 3). After establishing the mouse MCAO model, each group of mice underwent the same treatment protocol as described previously. 24 h later, the brain tissue samples were collected after cardiac perfusion, washed, weighed, dried, and weighed again, and the brain water content was calculated according to formula (5):

(5) $$\mathrm{Brain}\;\mathrm{water}\;\mathrm{content}(\%)=\frac{m_{1}-m_{2}}{m_{1}}\;\times \;100\%\;$$

where *m*_1_ is the wet weight of brain tissue and *m*_2_ is the dry weight of brain tissue.

### Behavioral evaluation

The mice were randomly divided into five groups (*n* = 6). After establishing the mouse MCAO model, each group of mice underwent the same treatment protocol as described previously. After 24 h, the mice regained consciousness, and the neurological deficit symptom score was conducted on the five groups of mice following the Longa standard: 0 indicated no signs of neurological impairment; 1 indicated a partial inability to extend the left forelimb due to adduction, 2 indicated turning toward the paralyzed side; 3 indicated falling to the opposite side of the injury; 4 indicated an inability to walk spontaneously or coma. The scoring process was conducted in a single-blind manner to ensure unbiased assessment, with raters unaware of the experimental animal groupings.

### Immunohistochemistry

The mice were randomly divided into five groups (*n* = 3). After establishing the mouse MCAO model, each group of mice underwent the same treatment protocol as described previously. After a duration of 24 h, following cardiac perfusion, brain tissue samples were collected, washed, and subsequently fixed with 4% paraformaldehyde for a specific duration. Immunohistochemical analysis was conducted to observe the expression levels of TLR4 markers in the brain tissue samples.

### TUNEL staining

TUNEL staining was performed using the TUNEL system kit for fluorescence measurement to assess apoptosis. The mice were randomly divided into five groups (*n* = 3) as mentioned earlier. After establishing the mouse MCAO model, each group of mice underwent the same treatment protocol as described previously for immunohistochemical staining. Brain tissues were fixed using formaldehyde and subsequently embedded in paraffin. A 5-µm-thick section of brain tissue was obtained and subjected to staining. Tunel-positive cells, which had fluorescent green nuclei due to the binding of DUtase to their DNA fragments, were considered apoptotic cells. All nuclei were stained with DAPI (blue). The merged image represents an overlay of TUNEL (green) and DAPI (blue) dyes.

### Cytotoxicity

Human neuroblastoma SHSY5Y cells were cultured in DMEM containing 10% fetal bovine serum and penicillin/streptomycin in a 5% CO_2_ humidified incubator at 37°C. The experiment was divided into three groups (*n* = 3): (i) Pte; (ii) BP-Pte@PDA; (iii) BP-Pte@PDA+NIR irradiation. To investigate the impact of NIR irradiation on cell viability, a CCK8 assay was conducted. SHSY5Y cells in the logarithmic growth phase were utilized, adjusted to a specific cell concentration, and seeded into 96-well plates at a density of 8 × 10^3^ cells/well. The cells were then cultured overnight in a CO_2_ incubator at 37°C. Subsequently, 100 μl/well of sample solutions at concentrations of 25, 50, 100, 150 and 200 μg/ml was added. Group 3 underwent NIR irradiation (808 nm, 1 W/cm^2^) for 10 min following the addition of the sample solutions and was further cultured for 24 h before the removal of the medium. Each well was cleaned with PBS three times, and a culture medium containing 10% CCK8 and 5% CO_2_ was added according to 120 μl/well, and cultured in a constant temperature incubator at 37°C for 2 h. The supernatant (100 μl/well) was transferred to a new 96-well plate, and the absorbance value at 450 nm was detected by enzymoleter. The cell survival rate was calculated according to formula (6):

(6) $$\mathrm{Cell}\;\mathrm{viability}\left(\%\right)=\frac{{\mathrm{OD}}_{2}-{\mathrm{OD}}_{0}}{{\mathrm{OD}}_{1}-{\mathrm{OD}}_{0}}\;\times \;100\;$$

where OD_0_, OD_1_ and OD_2_ are the background OD value, control OD value and experimental OD value respectively.

### Assessment of *in vivo* biocompatibility

The mice were randomly divided into four groups (*n* = 3): (i) Control; (ii) Pte; (iii) BP-Pte@PDA; (iv) BP-Pte@PDA+NIR irradiation. The designated solution was injected into the tail vein every day. After 7 days, blood samples were taken from the mice and their serum biochemical indexes were evaluated. Major organs (lung, kidney, spleen, heart and liver) were collected, fixed with 4% paraformaldehyde, paraffin-embedded, sectioned, and stained with hematoxylin and eosin (H&E) to evaluate tissue toxicity.

### Statistical analysis

SPSS 27.0 software was used for statistical analysis. The data were presented as mean ± standard deviation, and the difference between the two groups was analyzed using *t* test. *P*-values between 0.05 (*), 0.01 (**), 0.001 (***) and 0.0001 (****) were considered statistically significant differences.

## Results and discussions

### Characterization of BP preparations

**Figure 2.**
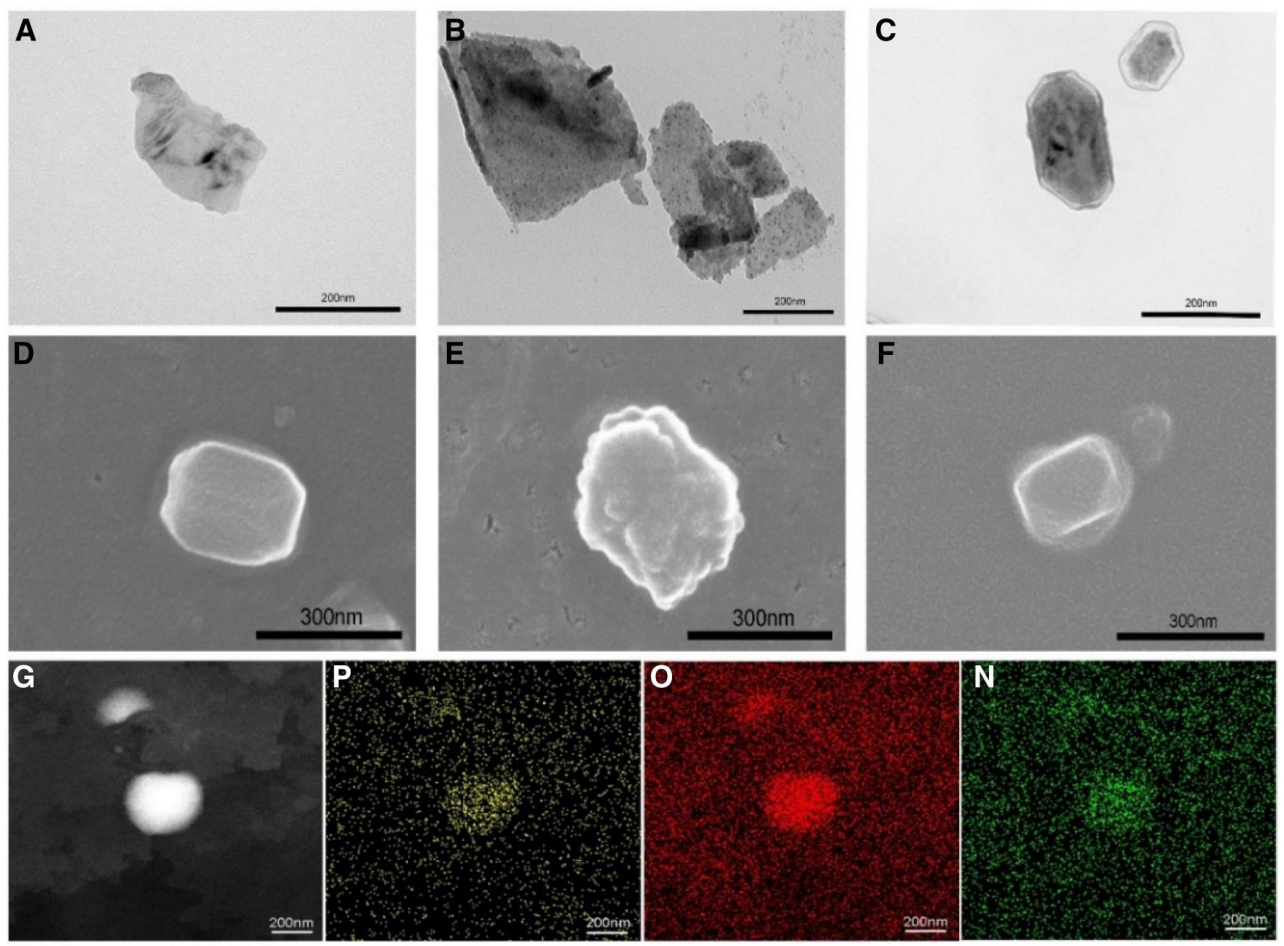
Morphology characterization of BP preparations. (**A**–**C**) TEM image of BP nanosheets, BP-Pte and BP-Pte@PDA. Scale bar: 200 nm. (**D**–**F**) SEM image of BP nanosheets, BP-Pte and BP-Pte@PDA. Scale bar: 300 nm. (**G**) The high-resolution scanning TEM image and elemental mapping of BP-Pte@PDA. Scale bar: 200 nm.

BP, BP-Pte and BP-Pte@PDA were analyzed by TEM, SEM, high-resolution scanning TEM, FTIR, UV-vis and Malvern laser particle size analyzer. The TEM and SEM image micrographs depicted distinct edges and layered structures in the three types of nanosheets, measuring between 200–300 nm (Figure 2A–F). A comparison between the TEM images of BP and BP-Pte revealed a considerable amount of black material covering the nanosheets in BP-Pte, signifying successful Pte loading onto the BP nanosheets (Figure 2A and B). Additionally, the TEM image of BP-Pte@PDA displayed a discernible PDA film (Figure 2C). It was observed that the degradation of the PDA film was complete under acidic and illumination conditions after the BP-Pte@PDA degraded (Supplementary Figure S1). Additionally, there was slight degradation of the BP nanosheets, and partial release of the Pte drug was evident. The distribution of P, O and N elements was evaluated by high-resolution scanning TEM. Bright field images and mapping analysis (Supplementary Figures S2A and S3A) confirmed the successful fabrication of pure BP nanosheets, without any surface coating, showing an overlap of P and O elements. In contrast, the oxygen (O) content in BP-Pte (Supplementary Figures S2B and S3B) is significantly higher than that in BP. This increase in O content in BP-Pte originates from Pte, indicating the presence of polyoxometalates (PO_x_) on its surface. This suggests successful loading of Pte onto BP, as evidenced by the high O content in BP-Pte compared to BP. Additionally, a small amount of nitrogen (N) in BP-Pte originates from the modification of mPEG-NH_2_ during the preparation process. Importantly, in the image corresponding to BP-Pte@PDA, the N content is significantly higher than in BP and BP-Pte, suggesting the successful modification of PDA on the surface of BP-Pte (Figure 2G and Supplementary Figures S3C and S3C).

**Figure 3.**
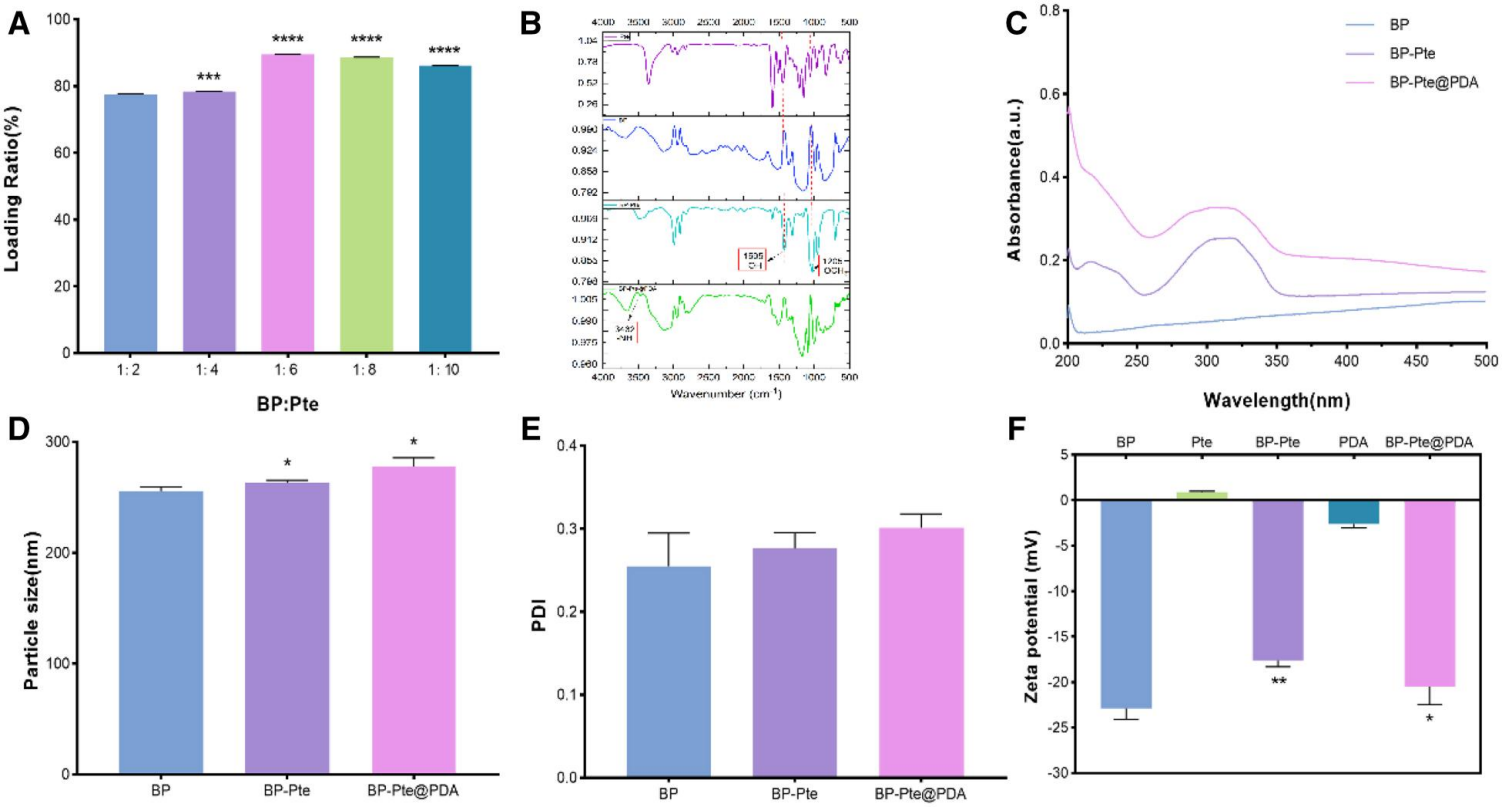
Properties characterization of BP preparations. (**A**) The drug loading ratio of BP and Pte with different mass ratios. (Compare to BP: Pte = 1:2 group, ***P <* *0.001, ****P <* *0.0001.) (**B**) FTIR spectra of BP, Pte, BP-Pte and BP-Pte@PDA. (**C**) The full wavelength UV spectra of BP, BP-Pte, and BP-Pte@PDA. (**D**) The particle size of BP, BP-Pte and BP-Pte@PDA. (Compare to BP group, *P <* *0.05.) (**E**) The PDI values of BP, BP-Pte and BP-Pte@PDA. (**F**) The zeta potential values of BP, BP-Pte and BP-Pte@PDA. (Compare to BP group, *P <* *0.05, **P <* *0.01).

According to the measurement of the drug loading ratio of different mass ratios of BP to Pte, the results showed a gradual increase in the drug loading ratio as the mass ratio increased. At mBP: mPte = 1:6, the drug loading ratio reached the highest value (89.496 ± 0.195%), and then tended to be stable (Figure 3A). The results show that in the process of preparing BP-Pte, when the quality of Pte is six times that of BP, the drug loading ratio can reach the highest. This maximizes the utilization of BP nanosheets, and ensures sufficient drug release for the optimal therapeutic effect.

The FTIR spectra of BP, Pte, BP-Pte and BP-Pte@PDA have been shown in (Figure 3B). The peak positions of BP-Pte are majorly consistent with that of Pte (-OH: 1915 cm^−1^, -OCH3:1205 cm^−1^), with a new peak (-NH, 3462 cm^−1^) appearing in the spectrum of BP-Pte@PDA, indicating that BP-Pte@PDA has been successfully prepared. The ultraviolet spectra of BP-Pte illustrate the successful loading of Pte (with a maximum absorbance at a corresponding wavelength of 307.4 nm) onto BP. Meanwhile, the full spectrum of BP-Pte@PDA demonstrates the successful modification of PDA onto BP-Pte (Figure 3C). These findings confirm the successful preparation of BP-Pte@PDA.

To investigate the change in particle size of the samples of BP, BP-Pte and BP-Pte@PDA were analyzed by a Malvern laser particle size analyzer. The average size of BP nanosheets measures 255.7 ± 3.9 nm, which increases to 263.1 ± 2.3 nm upon loading Pte, with some samples reaching 500 nm. Additionally, the average size of PDA-modified BP-Pte nanosheets further increases to 277.9 ± 7.8 nm (Figure 3D). These changes in particle size after Pte loading and PDA modification affirm the successful preparation of BP-Pte and BP-Pte@PDA. PDI values can reflect the homogeneity of nanomaterials, and the PDI values of BP, BP-Pte and BP-Pte@PDA are 0.255 ± 0.040, 0.276 ± 0.019 and 0.301 ± 0.017, respectively (Figure 3E). Compared with BP, the PDI values of BP-Pte and BP-Pte@PDA are higher, which is largely due to the uneven size caused by BP-Pte stacking. PDA within BP-Pte@PDA tends to form nanospheres, causing aggregation and resulting in uneven dispersion, hence contributing to a larger PDI value.

To further investigate how Pte and PDA affect the stability of BP, the zeta potential values of BP, Pte, BP-Pte, PDA and BP-Pte@PDA were measured. The results show that the addition of Pte changes the zeta value from −22.9 ± 1.2 mV (BP) to −17.6 ± 0.7 mV (BP-Pte). Since the magnitude of the zeta potential of Pte is 0.9 ± 0.1 mV, the absolute zeta potential value of BP nanosheet loaded with Pte becomes lower. This suggests that the inclusion of Pte compromised the stability of BP. When BP-Pte is coated with PDA, the zeta potential of BP-Pte decreases to −20.5 ± 2.0 mV due to the presence of hydroxyl group in the PDA (zeta value of hydroxyl group is −2.6 ± 0.4 mV) (Figure 3F). With the addition of Pte, the absolute zeta potential value notably decreased. Following the PDA modification, there was a considerable increase in the absolute zeta potential value, indicating an enhancement in the stability of BP-Pte due to the PDA modification. These findings confirm the successful loading of Pte onto BP nanosheets as well as the coating of the PDA layer onto the surface of BP-Pte, thereby resulting in the successful fabrication of the BP-Pte@PDA drug delivery system.

### Photothermal performance evaluation

**Figure 4.**
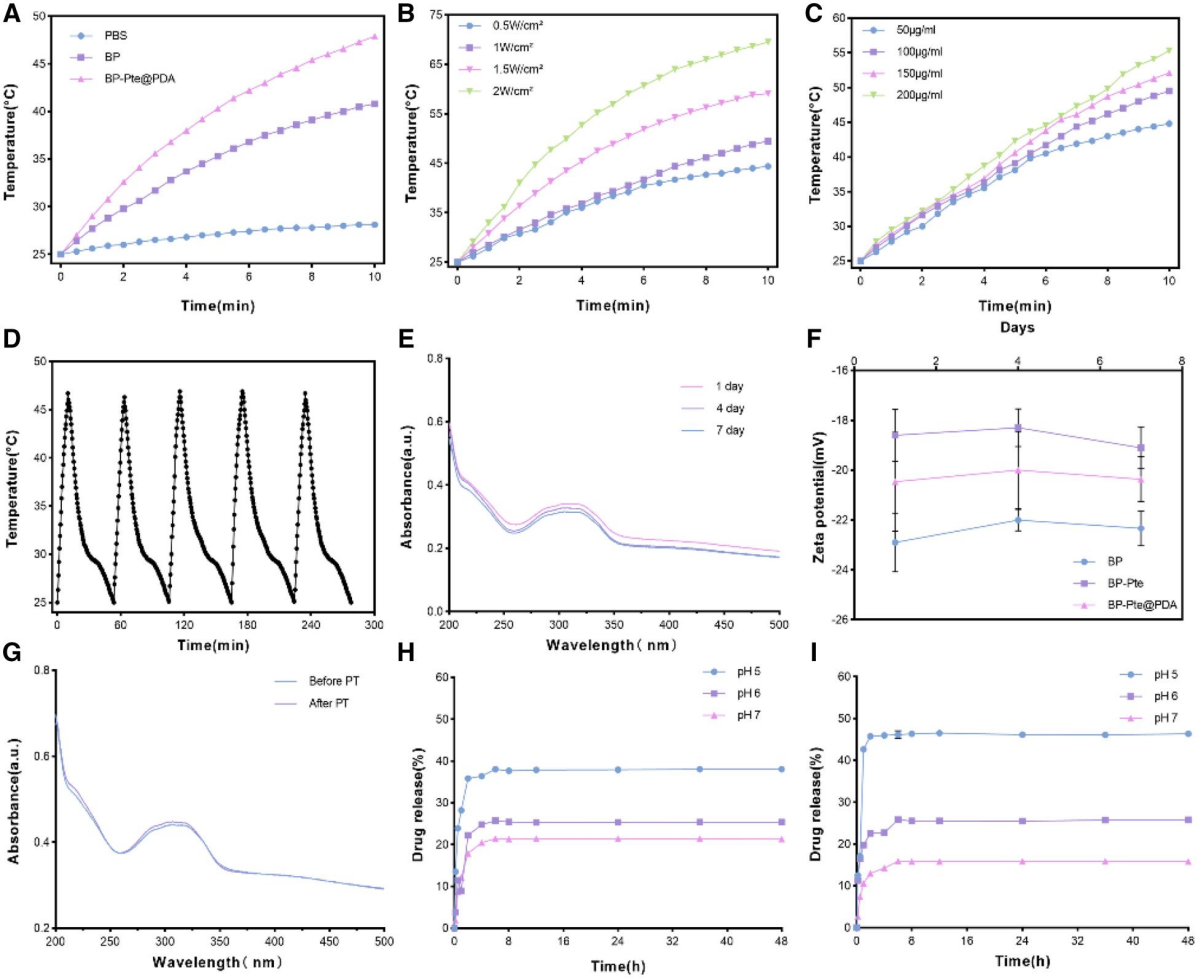
Evaluation of photothermal properties, stability, and drug release. (**A**) Temperature change of PBS, BP and BP-Pte@PDA under NIR (1 W/cm^2^) irradiation. (**B**) 100 μg/ml BP-Pte@PDA’s temperature change under NIR irradiation with different powers. (**C**) Temperature change curves of different concentrations of BP-Pte@PDA under NIR (1 W/cm^2^) irradiation. (**D**) Five cycles of heating and cooling of BP-Pte@PDA. (**E**) The wavelength change curve of BP-Pte@PDA solution in 7 days. (**F**) The change of zeta potential value of BP-Pte@PDA solution within 7 days. (**G**) The wavelength change of BP-Pte@PDA before and after NIR (808 nm, 1 W/cm^2^) irradiation. (**H**) *In vitro* drug release rate of BP-Pte@PDA without NIR irradiation. (**I**) *In vitro* drug release rate of BP-Pte@PDA with NIR irradiation.

To further investigate the photothermal effects of BP formulations, the impact of NIR on varying concentrations of BP and BP-Pte@PDA was assessed. The findings indicated that temperatures increased over time for 100 μg/ml PBS, BP and BP-Pte@PDA. BP-Pte@PDA displayed a significant temperature rise, rising from 25°C to 47.9°C (Figure 4A). Moreover, under different NIR powers, the temperatures of 100 μg/ml BP and BP-Pte@PDA increased progressively with time (Figure 4B and Supplementary Figure S4A). At a power density of 2.0 W/cm^2^, temperatures spiked significantly, reaching peaks of 64.1 and 69.5°C, respectively (Figure 4B). With the increase of the concentration of the solution, the temperature also increases (Figure 4C and Supplementary Figure S4B), and the temperature increases significantly when the concentration is 200 μg/ml, reaching 48 and 55.3°C (Figure 4C). During the five irradiation cycles, the peak temperatures of BP and BP-Pte@PDA in the first cycle were 40.8°C and 46.7°C, respectively. Interestingly, in subsequent cycles, BP-Pte@PDA exhibited minimal variance in its maximum temperatures (Figure 4D and Supplementary Figure S4C). These results highlight the favorable photothermal characteristics of both BP and BP-Pte@PDA. BP-Pte@PDA demonstrates superior photothermal efficiency compared to BP alone, which is likely due to the increased photothermal properties imparted by PDA.

### Stability evaluation

The interaction between BP nanosheets and drugs occurs through the lone pair electrons on their surface, facilitating electrostatic adsorption. However, the BP surface electrons have an affinity for oxygen, leading to BP nanosheet oxidation and consequent instability. The bonding between BP nanosheets and Pte relies on electrostatic adsorption, while dopamine hydrochloride undergoes autopolymerization in a mild alkaline environment, forming PDA that coats the BP-Pte surface. The coating of PDA film can enhance the stability of the whole system. By observing the full spectrum changes of BP, BP-Pte and BP-Pte@PDA over 1, 4 and 7 days, it was evident that the absorbance of BP and BP-Pte notably decreased (Supplementary Figure S5A and B). Conversely, the absorbance of BP-Pte@PDA formulation showed minimal change, suggesting that the incorporation of PDA contributed to the enhanced stability of the entire system (Figure 4E). Based on the change curve of their zeta potential values, while BP and BP-Pte experienced a considerable amplitude change of approximately 0.6 and 0.5 mV, respectively, BP-Pte@PDA demonstrated a minimal change in zeta potential value (∼0.1 mV), that too with a smaller amplitude (Figure 4F). These findings highlight the robust stability of BP-Pte@PDA.

Photothermal stability can be determined by the change in maximum temperature and absorbance after five cycles of irradiation. Following five cycles, it was found that the maximum temperature of BP decreased with the increase in the cycle, from 40.5°C to 39.0°C (Supplementary Figure S4C), and the ultraviolet absorbance of BP also decreased significantly as evident from the full-wavelength spectrum (Supplementary Figure S5C). In contrast, throughout the five illumination cycles, the maximum temperature of BP-Pte@PDA remains consistently around 46.7°C, showing no significant variation (Figure 3D and G). Similarly, its absorbance undergoes a minimal decrease. This stability can be attributed to the Pte load and PDA modification, which reduce the extent of absorbance reduction, ensuring a more stable temperature profile. These characteristics indicate the robust photothermal stability of BP-Pte@PDA.

### Analysis of *in vitro* drug release

In the 48-h *in vitro* drug release experiment, the results (Figure 4H and I and Supplementary Table S1) showed that the 48-h *in vitro* drug release rate of the BP-Pte@PDA+NIR irradiation group was higher than that of the BP-Pte@PDA group at a pH value of 5. The drug release rates of the drug delivery system at pH 5, 6 and 7 under NIR irradiation were 46.529 ± 0.502%, 25.806 ± 0.160% and 15.893 ± 0.010%. The drug release rates without NIR irradiation were 38.061 ± 0.035%, 25.735 ± 0.043% and 21.455 ± 0.021%, respectively. These findings suggest that the *in vitro* release rate of Pte from the drug delivery system is responsive to pH *in vitro*, with an increased release rate observed in weakly acidic pH environments. This release behavior is attributed to the degradation of PDA in acidic conditions. Additionally, following NIR irradiation the significant thermal effect of BP leads to the conversion of a large amount of light energy into heat energy, resulting in a local temperature rise. This acceleration in the degradation of BP itself facilitates the release of more Pte into the body. As the temperature decreases, the drug release returns to baseline levels, resulting in a gradual recovery of the release curve. These results indicate that BP-Pte@PDA demonstrates optimal drug release under NIR irradiation at a pH of 5.

### Assessment of BBB permeability

**Figure 5.**
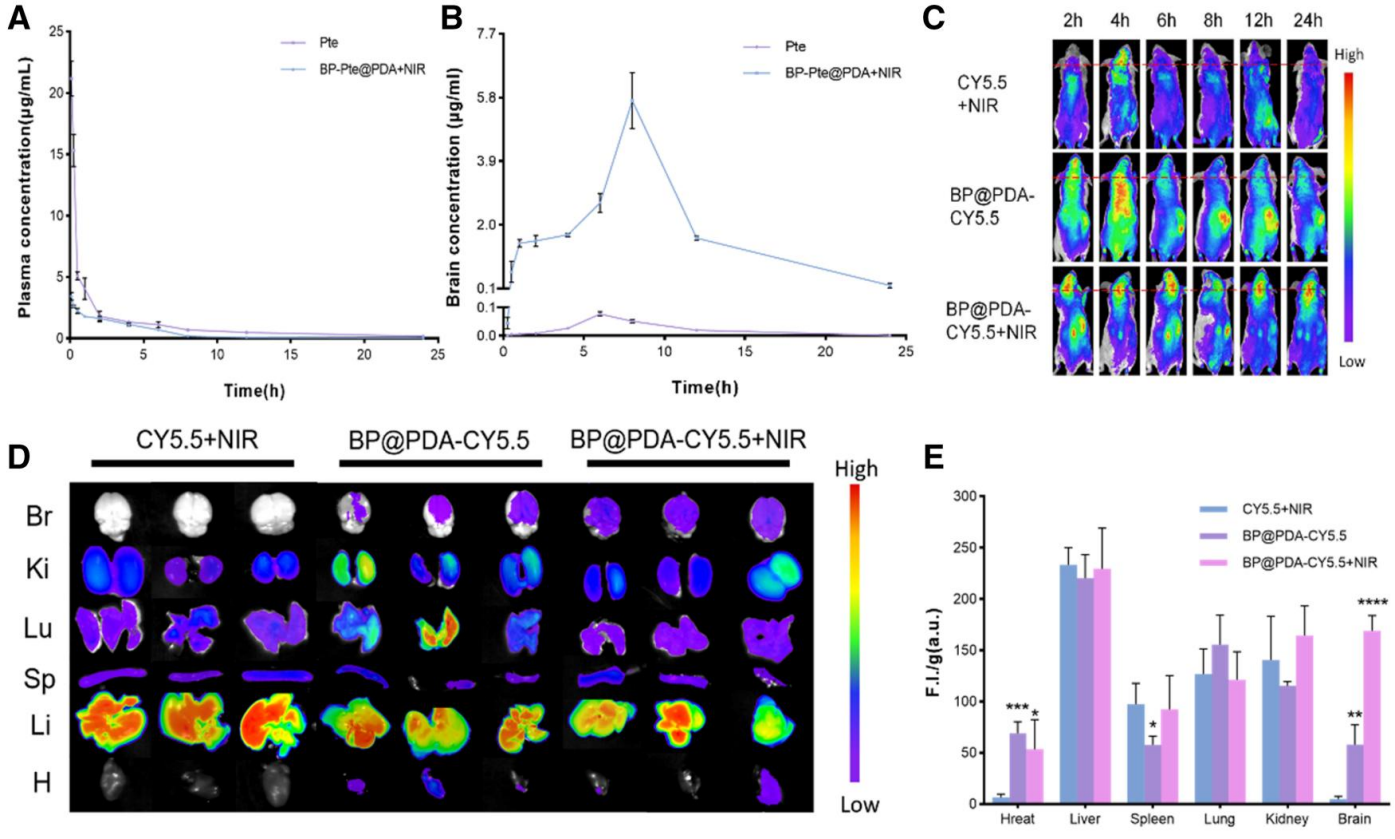
Evaluation of BBB permeability. (**A**) Plasma concentration-time curve of Pte following administration of Pte and BP-Pte@PDA+NIR (*n* = 3). (**B**) Pte concentration-time curve in brain tissue following administration of Pte and BP-Pte@PDA+NIR (*n* = 3). (**C**) *In vivo* whole-body fluorescence imaging of mice from 2 to 24 h (*n* = 1). (**D**) Fluorescence imaging was performed on the major organs of the mice treated for 6 h (*n* = 3). (**E**) Cy5.5 levels in major organs (*n* = 3). (Compare to CY5.5+NIR group, *P <* *0.05, **P <* *0.01, ***P <* *0.001, ****P <* *0.0001.)

The results of UPLC analysis can be used to analyze the behavior of BP-Pte@PDA *in vivo*. The outcomes from the plasma drug concentration–time curve and its parameters indicated significant differences between BP-Pte@PDA+NIR and Pte in terms of *T*_1/2_ (2.527 ± 0.785 vs 7.965 ± 0.547 h) and MRT_0-__*t*_ (3.721 ± 0.105 vs 4.676 ± 0.056 h) with BP-Pte@PDA+NIR showing 3.15 times and 1.26 times variation, respectively. However, the *C*_max_ of Pte (21.155 ± 1.427 μg/ml) was 6.16 times that of BP-Pte@PDA+NIR (7.965 ± 0.547 μg/ml), indicating that Pte alone was more easily absorbed by the blood (Figure 5A and Supplementary Table S2). The concentration–time curve of the cerebral drug after intravenous administration was further measured, and the pharmacokinetic parameters were calculated. The results showed that cerebral Pte concentration reached its peak after BP-Pte@PDA 8 h of NIR irradiation, and the *C*_max_ of BP-Pte@PDA + NIR group was 5.701 ± 0.844 μg/ml. The *C*_max_ of the Pte group was 0.077 ± 0.008 μg/ml, in contrast, indicating that BP-Pte@PDA completely released Pte in the brain under NIR irradiation, and pure Pte was more easily absorbed into the blood (Figure 5B and Supplementary Table S3). The robust pharmacokinetic findings affirmed the ability of BP-Pte@PDA to breach the BBB, accessing brain tissue and effectively releasing Pte.

To evaluate in depth the ability of BP@PDA to target the brain *in vivo*, fluorescence imaging of live and isolated mouse tissues was performed using the LB983 *in vivo* imaging system. After the successful establishment of the mouse MCAO model, the specified solution was given. The outcomes revealed that in groups administered with BP@PDA-CY5.5 and BP@PDA-CY5.5+NIR irradiation, the fluorescence from was detected in brain tissue after 2 h, peaking at 6 h (Figure 5C and Supplementary Figure S6). The fluorescence intensity in brain tissue under BP@PDA-CY5.5+NIR irradiation was notably higher than that without NIR irradiation. This suggests that NIR irradiation further boosted the accumulation of BP@PDA in the brain, confirming its enhanced BBB permeability. In addition, fluorescence levels in the heart and spleen were lower than those in the lungs, kidneys, and liver, indicating that BP@PDA preparation is easily captured by the reticuloendothelial system-related organs before kidney clearance (Figure 5D and E). These results indicated that BP-Pte@PDA preparation could effectively penetrate the BBB, and could reach the ischemic region and release drugs.

### Evaluation of pharmacodynamics *in vivo*

**Figure 6.**
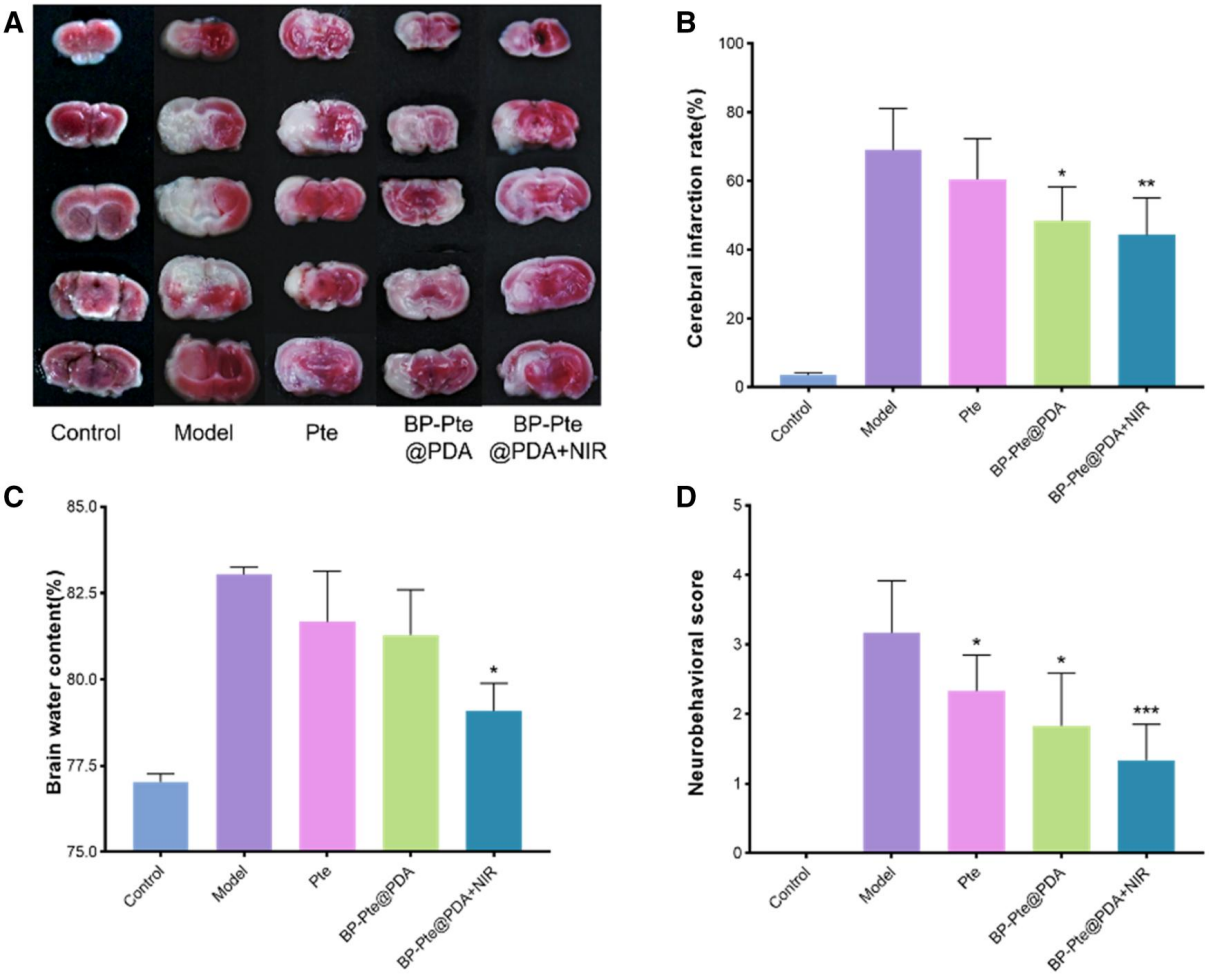
Evaluation of *in vivo* pharmacodynamics parameters in MCAO mice in different administration groups. (**A**) The TTC staining diagram of different treatment groups (*n* = 3). (**B**) The cerebral infarction rate of different treatment groups (*n* = 3). (**C**) The brain water content of different treatment groups (*n* = 3). (**D**) The neurobehavioral score of different treatment groups (*n* = 6). (Compare to control group, *P <* *0.05, **P <* *0.01, ***P <* *0.001.)

To evaluate the potential of BP-Pte@PDA for the treatment of IS, different drugs were injected intravenously in a mouse model of MCAO, followed by 808 nm laser irradiation of a specific group. After 24 h, the infarct rate, water content, behavior, immunohistochemistry and tunnel staining were analyzed. The results of TTC staining and cerebral infarction rate showed that control mice did not show any signs of infarction. After the MCAO operation, the infarction rate of mice was 69.070 ± 12.050%, indicating that the MCAO model was successfully established. In contrast, when the mice received Pte, BP-Pte@PDA and BP-Pte@PDA with NIR laser irradiation, the infarct percentage decreased to 60.420 ± 11.982%, 48.401 ± 9.923% and 44.282 ± 10.846%, while the infarct size decreased significantly in the BP-Pte@PDA+NIR irradiation group (Figure 6A and B). The results showed that the brain water content of the control group was 77.039 ± 0.221%, while that of the MCAO model group was 83.047 ± 0.196%. There was a significant difference between the MCAO model group and the sham operation group, indicating that the modeling was successful. In contrast, the water content of the Pte and BP-Pte@PDA groups is 81.290 ± 1.305%, and that of the BP-Pte@PDA group under NIR irradiation is 79.090 ± 0.796%, which is close to the value of the control group and the MCAO model group (Figure 6C). An analysis of the behavioral scores shows that the score of the MCAO model group is 3.167 ± 0.753, while those of Pte and BP-Pte@PDA are 2.333 ± 0.516 and 1.833 ± 0.753, which are significantly different from those of the MCAO model group. In contrast, the score of BP-Pte@PDA+NIR group is 1.333 ± 0.516, which is significantly different from that of the MCAO model group (Figure 6D). These results indicate that BP-Pte@PDA has a good therapeutic effect on IS under NIR irradiation.

**Figure 7.**
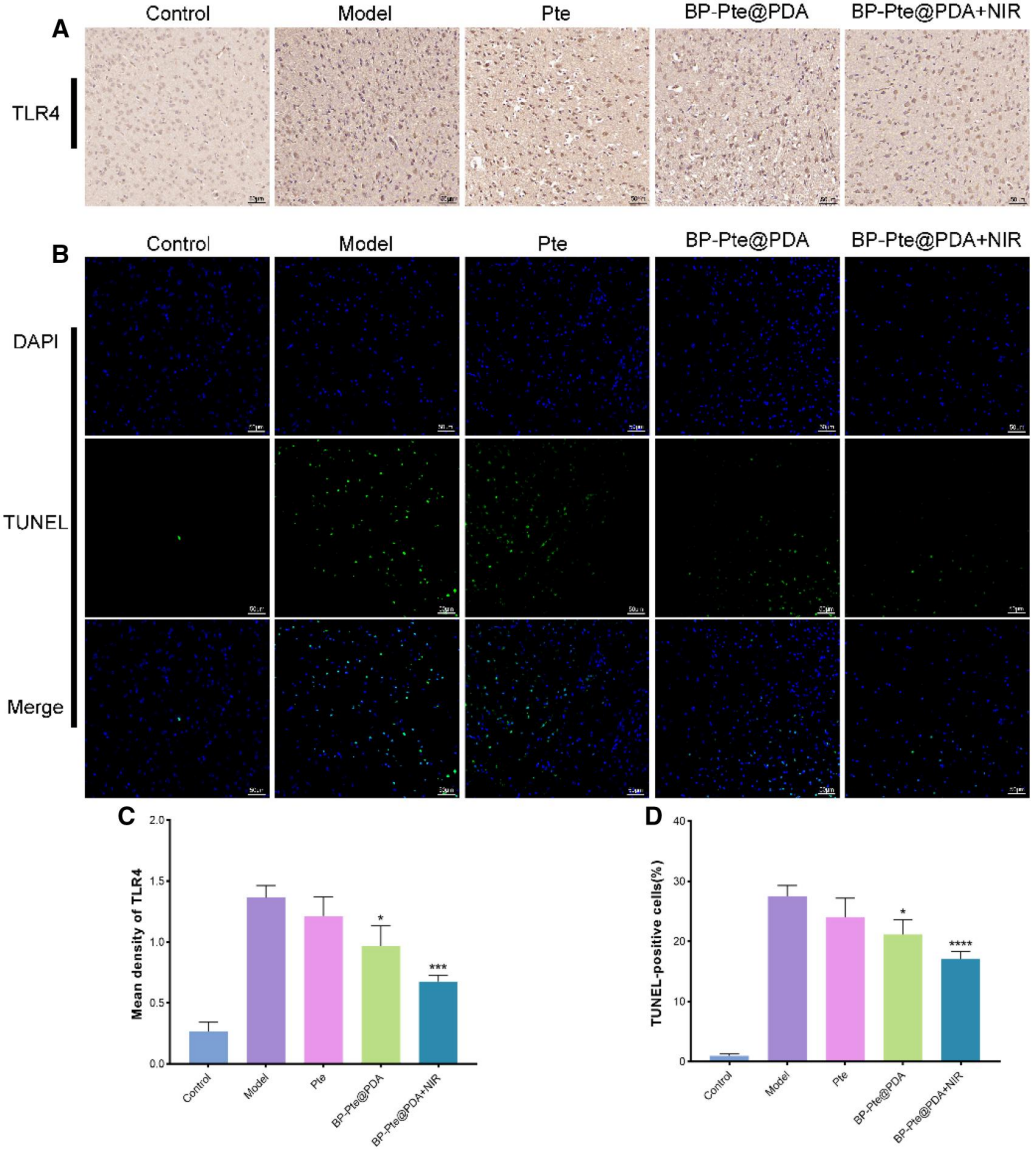
Effects of each group on the expression of TLR4 inflammatory factor and the number of TUNEL positive cells in MCAO mice. (**A**) Immunohistochemical staining of TLR4 in brain tissue (*n* = 3). Scale bar: 50 μm. (**B**) Representative TUNEL-staining brain sections in different groups(*n* = 3). Scale bar: 50 μm. (**C**) Quantitative analysis of TLR4 content in different groups (*n* = 3). (Compare to control group, *P <* *0.05, ***P <* *0.001.) (**D**) Quantitative analysis of apoptotic cells in different groups (*n* = 3). (Compare to control group, *P <* *0.05, ****P <* *0.0001.)

Toll-like receptors (TLRs) are a group of innate immune receptors that identify pathogen-associated molecular patterns, thus activating pertinent signal transduction pathways and contributing significantly to inflammatory responses. Among the TLRs present in the mammalian central nervous system, TLR4 is predominantly expressed in microglia and is involved in the mediation of neuroinflammatory diseases [35]. In several animal stroke models, TLR2 or TLR4 deletion resulted of a significant reduction in infarct size, suggesting that activation of TLR2 and TLR4 during IS exacerbates injury [36, 37]. In IS, activation of the TLR4/NF-kB pathway induces the polarization of microglia towards the M1 phenotype [38] and triggers downstream signaling via the MyD88 adapter, resulting in the production of pro-inflammatory cytokines [39]. Zhang *et al.* [40] discovered that vagal nerve stimulation therapy can mitigate brain injury during the acute phase of stroke by suppressing the TLR4/MyD88/NF-kB pathway in microglia and modulating microglial polarization. Through animal and cellular experiments, Xu *et al.* [41] demonstrated that Pte ameliorates cognitive dysfunction in vascular dementia models by attenuating TLR4-mediated inflammatory responses. In this study, the expression of the TLR4 factor was detected in animals, and the immunohistochemical results (Figure 7A and C) of the brain tissues of each group showed that the expression of TLR4 was very high in the model group. In contrast, the expression of TLR4 in the BP-Pte@PDA group and BP-Pte@PDA+NIR group was lower. The expression of TLR4 in the BP-Pte@PDA+NIR group exhibited a notable difference compared to that in the model group. This suggests that the BP-Pte@PDA drug delivery system has the potential to exert anti-inflammatory effects by suppressing the release of the TLR4 factor. Consequently, it may yield favorable outcomes in the treatment of IS.

It has been reported that apoptosis and autophagy contribute to the pathophysiology of IS [42, 43], highlighting the importance of inhibiting these processes to enhance patient outcomes [44, 45]. Liu *et al.* [46] demonstrated that Pte effectively reduced neuronal apoptosis in the vicinity of the infarct area and enhanced the survival rate of neuronal cells *in vitro*. In this study, TUNEL staining was employed to assess MCAO-induced apoptosis of brain cells in each group. The findings (Figure 7B and D) revealed a significant increase in the number of apoptotic cells emitting green fluorescence in the MCAO group compared to the control group. Compared with the MCAO group, the apoptosis rate of the BP-Pte@PDA group and BP-Pte@PDA+NIR group differed significantly, and the apoptosis of neuronal cells was significantly inhibited. These results suggest that BP-Pte@PDA treatment significantly inhibits apoptosis. Overall, the BP-Pte@PDA drug delivery system demonstrates the ability to suppress the expression of inflammatory cytokines and prevent cell apoptosis.

### Blood compatibility and cytotoxicity assessment

**Figure 8.**
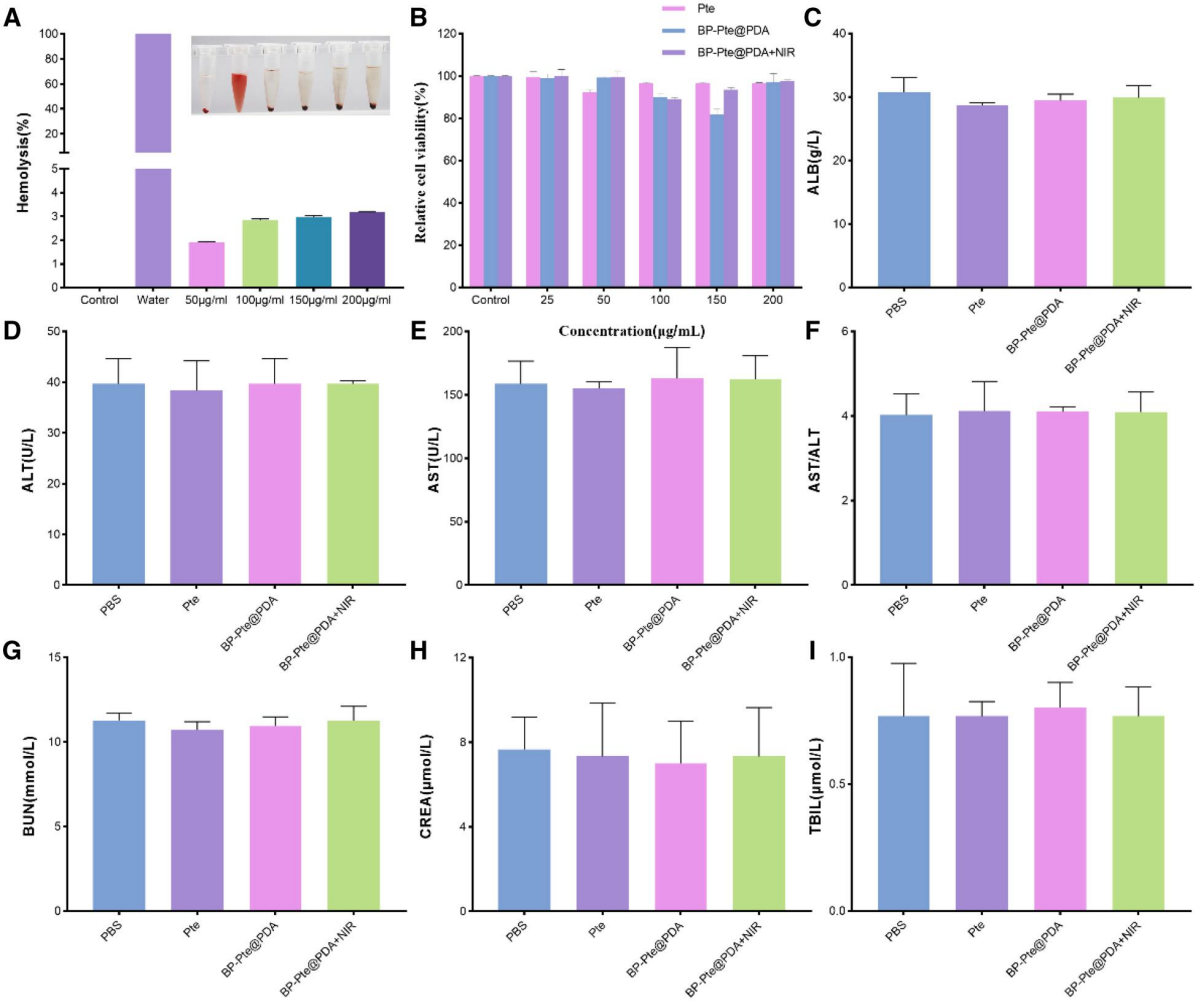
Safety evaluation. (**A**) The hemolysis rate of different concentrations of BP-Pte@PDA solution (*n* = 3). (**B**) *In vitro* cell viability assay of different administration groups (*n* = 3). (**C**–**I**) the serum biochemical indicators (ALB, ALT, AST, AST/ALT, BUN, CREA, TBIL) in different groups of treatment (*n* = 3).

Before conducting *in vivo* experiments, it is necessary to determine whether BP-Pte@PDA has the potential to induce hemolysis. The blood compatibility of different concentrations (50, 100, 150, 200 μg/ml) of BP-Pte@PDA was determined using a hemolysis test, with pure water as the positive control and 0.9% normal saline as the negative control. The results showed that the supernatant of the positive control group was bright red with no precipitation at the bottom, indicating the destruction of red blood cells and the occurrence of a severe hemolytic reaction. However, the supernatant of the negative control group and BP-Pte@PDA with different concentrations appeared clear and transparent, with red blood cell precipitation at the bottom. The hemolysis rate of BP-Pte@PDA was all below 5%, indicating the absence of a hemolytic reaction and confirming its suitability for intravenous injection (Figure 8A).

The cytotoxicity assessment of Pte, BP-Pte@PDA and BP-Pte@PDA under NIR irradiation was conducted using the CCK8 method to measure SHSY5Y cell viability when exposed to different concentrations of these solutions. The findings indicated that even at a high drug concentration of 200 μg/ml, the cell viability remained above 80% in each group. This indicates the low cytotoxicity and relative safety of these formulations (Figure 8B).

### Evaluation of biocompatibility *in vivo*

**Figure 9.**
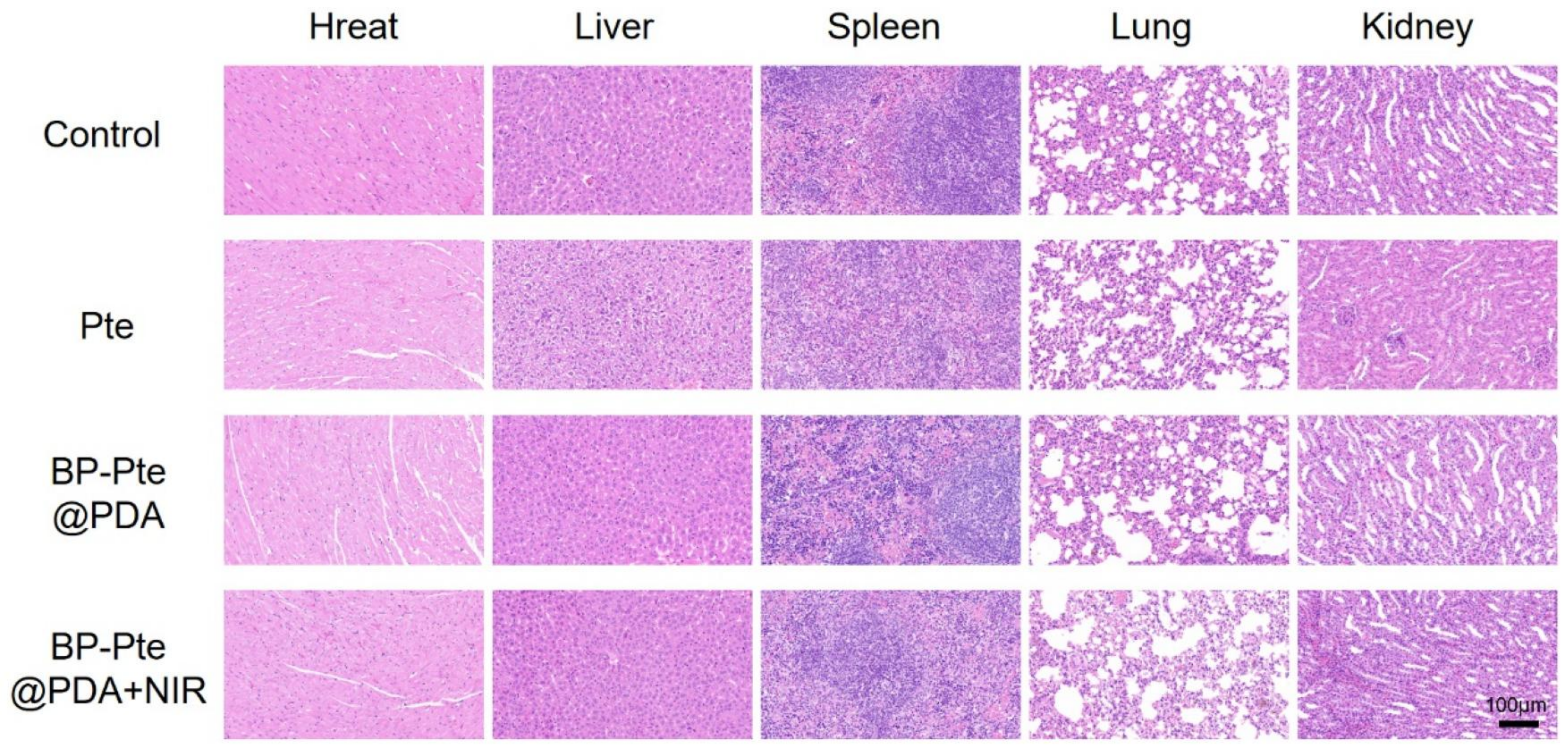
*In vivo* biocompatibility analysis. H&E staining of heart, liver, spleen, lung and kidney tissue sections from animals in the indicated treatment groups (*n* = 3). Scale bar: 100 μm.

After a 7-day dosing period, blood samples from mice were analyzed for routine serum enzyme levels (ALT, AST, ALB, BUN, CREA, TBIL). No significant differences were observed in these indicators across the various administration groups, suggesting no apparent renal, liver, or hematological toxicity induced by the treatments (Figure 8C–I). Furthermore, H&E staining revealed no substantial tissue damage in major organs (Figure 9 and Supplementary Figure S7), and there were no notable changes in body weight. These findings support the *in vivo* biocompatibility of BP-Pte@PDA, suggesting its suitability as a novel nanomaterial preparation for treating IS.

## Conclusions

This study designed a novel BP-Pte@PDA drug delivery system, which demonstrated a promising therapeutic effect on IS. The experimental findings highlight the drug delivery system’s robust photothermal response, stability, pH and NIR responsivity, biocompatibility and notable therapeutic efficacy against IS. NIR irradiation enhances BP-Pte@PDA’s *in vivo* BBB permeability, leading to Pte release in the IS environment. Consequently, it diminishes cerebral infarction size, lowers cerebral water content, improves neurological deficits, reduces the TLR4 inflammatory factor expression and inhibits cell apoptosis. The excellent stability, therapeutic efficacy and biocompatibility of BP-Pte@PDA demonstrate its potential as an ideal drug delivery approach for treating brain diseases. Overall, these findings offer promising applications in various medical domains.

## Supplementary Material

rbae046_Supplementary_Data

## References

[1] Liu X , Li T, Diao S, Cai X, Kong Y, Zhang L, Wang Z, Li R, Zhou Y, Fang Q. The global burden of cerebral small vessel disease related to neurological deficit severity and clinical outcomes of acute ischemic stroke after IV rt-PA treatment. Neurol Sci 2019;40:1157–66. 30830567 10.1007/s10072-019-03790-x

[2] Wang Y-J , Li Z-X, Gu H-Q, Zhai Y, Zhou Q, Jiang Y, Zhao X-Q, Wang Y-L, Yang X, Wang C-J, Meng X, Li H, Liu L-P, Jing J, Wu J, Xu A-D, Dong Q, Wang D, Wang W-Z, Ma X-D, Zhao J-Z; China Stroke Statistics Writing Committee. China stroke statistics: an update on the 2019 report from the national center for healthcare quality management in neurological diseases, China national clinical research center for neurological diseases, the Chinese stroke association, national center for chronic and non-communicable disease control and prevention, Chinese center for disease control and prevention and institute for global neuroscience and stroke collaborations. Stroke Vasc Neurol 2022;7:415–50. 35443985 10.1136/svn-2021-001374 PMC9614174

[3] Shehadah A , Franklin GM, Benson RT. Global disparities in stroke and why we should care. Neurology 2016;87:450–1. 27371491 10.1212/WNL.0000000000002925

[4] Cozene B , Sadanandan N, Gonzales-Portillo B, Saft M, Cho J, Park YJ, Borlongan CV. An extra breath of fresh air: hyperbaric oxygenation as a stroke therapeutic. Biomolecules 2020;10:1279. 32899709 10.3390/biom10091279 PMC7563917

[5] Hatem SM , Saussez G, Della Faille M, Prist V, Zhang X, Dispa D, Bleyenheuft Y. Rehabilitation of motor function after stroke: a multiple systematic review focused on techniques to stimulate upper extremity recovery. Front Hum Neurosci 2016;10:442. 27679565 10.3389/fnhum.2016.00442 PMC5020059

[6] Chamorro Á , Dirnagl U, Urra X, Planas AM. Neuroprotection in acute stroke: targeting excitotoxicity, oxidative and nitrosative stress, and inflammation. Lancet Neurol 2016;15:869–81. 27180033 10.1016/S1474-4422(16)00114-9

[7] Venkat P , Shen Y, Chopp M, Chen J. Cell-based and pharmacological neurorestorative therapies for ischemic stroke. Neuropharmacology 2018;134:310–22. 28867364 10.1016/j.neuropharm.2017.08.036 PMC5832535

[8] Khoshnam SE , Winlow W, Farzaneh M, Farbood Y, Moghaddam HF. Pathogenic mechanisms following ischemic stroke. Neurol Sci 2017;38:1167–86. 28417216 10.1007/s10072-017-2938-1

[9] Lin X , Li N, Tang H. Recent advances in nanomaterials for diagnosis, treatments, and neurorestoration in ischemic stroke. Front Cell Neurosci 2022;16:885190. 35836741 10.3389/fncel.2022.885190 PMC9274459

[10] Savitz SI , Fisher M. Future of neuroprotection for acute stroke: in the aftermath of the SAINT trials. Ann Neurol 2007;61:396–402. 17420989 10.1002/ana.21127

[11] Kaushik A , Jayant RD, Bhardwaj V, Nair M. Personalized nanomedicine for CNS diseases. Drug Discov Today 2018;23:1007–15. 29155026 10.1016/j.drudis.2017.11.010 PMC6897362

[12] Ghosal K , Sarkar K. Biomedical applications of graphene nanomaterials and Beyond. ACS Biomater Sci Eng 2018;4:2653–703. 33434995 10.1021/acsbiomaterials.8b00376

[13] Pérez-Herrero E , Fernández-Medarde A. Advanced targeted therapies in cancer: drug nanocarriers, the future of chemotherapy. Eur J Pharm Biopharm 2015;93:52–79. 25813885 10.1016/j.ejpb.2015.03.018

[14] Mohammadi-Samani S , Taghipour B. PLGA micro and nanoparticles in delivery of peptides and proteins; problems and approaches. Pharm Dev Technol 2015;20:385–93. 24483777 10.3109/10837450.2014.882940

[15] Dobrovolskaia MA. Dendrimers effects on the immune system: insights into toxicity and therapeutic utility. Curr Pharm Des 2017;23:3134–41. 28294045 10.2174/1381612823666170309151958

[16] Yang X , Liu G, Shi Y, Huang W, Shao J, Dong X. Nano-black phosphorus for combined cancer phototherapy: recent advances and prospects. Nanotechnology 2018;29:222001. 29504512 10.1088/1361-6528/aab3f0

[17] Anju S , Ashtami J, Mohanan PV. Black phosphorus, a prospective graphene substitute for biomedical applications. Mater Sci Eng C Mater Biol Appl 2019;97:978–93. 30678986 10.1016/j.msec.2018.12.146

[18] Tao W , Zhu X, Yu X, Zeng X, Xiao Q, Zhang X, Ji X, Wang X, Shi J, Zhang H, Mei L. Black phosphorus nanosheets as a robust delivery platform for cancer theranostics. Adv Mater 2017;29:10.1002/adma.201603276. PMC5205548 27797119

[19] Xiong S , Li Z, Liu Y, Wang Q, Luo J, Chen X, Xie Z, Zhang Y, Zhang H, Chen T. Brain-targeted delivery shuttled by black phosphorus nanostructure to treat Parkinson’s disease. Biomaterials 2020;260:120339. 32861017 10.1016/j.biomaterials.2020.120339

[20] Liu H , Neal AT, Zhu Z, Luo Z, Xu X, Tománek D, Ye PD. Phosphorene: an unexplored 2D semiconductor with a high hole mobility. ACS Nano 2014;8:4033–41. 24655084 10.1021/nn501226z

[21] Chen X , Zhang S, Liu J, Ren M, Xing D, Qin H. Controlling dielectric loss of biodegradable black phosphorus nanosheets by iron-ion-modification for imaging-guided microwave thermoacoustic therapy. Biomaterials 2022;287:121662. 35797855 10.1016/j.biomaterials.2022.121662

[22] Li Z , Yu Y, Zeng W, Ding F, Zhang D, Cheng W, Wang M, Chen H, Pan G, Mei L, Zeng X, Gao N. Mussel-inspired ligand clicking and ion coordination on 2D black phosphorus for cancer multimodal imaging and therapy. Small 2022;18:e2201803. 35616079 10.1002/smll.202201803

[23] Chen Y , Ai K, Liu J, Ren X, Jiang C, Lu L. Polydopamine-based coordination nanocomplex for T1/T2 dual mode magnetic resonance imaging-guided chemo-photothermal synergistic therapy. Biomaterials 2016;77:198–206. 26606445 10.1016/j.biomaterials.2015.11.010

[24] Chang D , Gao Y, Wang L, Liu G, Chen Y, Wang T, Tao W, Mei L, Huang L, Zeng X. Polydopamine-based surface modification of mesoporous silica nanoparticles as pH-sensitive drug delivery vehicles for cancer therapy. J Colloid Interface Sci 2016;463:279–87. 26550786 10.1016/j.jcis.2015.11.001

[25] Tsai H-Y , Ho C-T, Chen Y-K. Biological actions and molecular effects of resveratrol, pterostilbene, and 3′-hydroxypterostilbene. J Food Drug Anal 2017;25:134–47. 28911531 10.1016/j.jfda.2016.07.004 PMC9333438

[26] Kapetanovic IM , Muzzio M, Huang Z, Thompson TN, McCormick DL. Pharmacokinetics, oral bioavailability, and metabolic profile of resveratrol and its dimethylether analog, pterostilbene, in rats. Cancer Chemother Pharmacol 2011;68:593–601. 21116625 10.1007/s00280-010-1525-4 PMC3090701

[27] Guo Y , Zhang L, Li F, Hu C-P, Zhang Z. Restoration of sirt1 function by pterostilbene attenuates hypoxia-reoxygenation injury in cardiomyocytes. Eur J Pharmacol 2016;776:26–33. 26921129 10.1016/j.ejphar.2016.02.052

[28] Khan MM , Badruddeen N, Ahmad U, Akhtar J, Khan MI, Khan MF. Cerebroprotective effect of pterostilbene against global cerebral ischemia in rats. Heliyon 2021;7:e07083. 34095578 10.1016/j.heliyon.2021.e07083 PMC8150920

[29] Naik B , Nirwane A, Majumdar A. Pterostilbene ameliorates intracerebroventricular streptozotocin induced memory decline in rats. Cogn Neurodyn 2017;11:35–49. 28174611 10.1007/s11571-016-9413-1 PMC5264756

[30] Chang J , Rimando A, Pallas M, Camins A, Porquet D, Reeves J, Shukitt-Hale B, Smith MA, Joseph JA, Casadesus G. Low-dose pterostilbene, but not resveratrol, is a potent neuromodulator in aging and Alzheimer’s disease. Neurobiol Aging 2012;33:2062–71. 21982274 10.1016/j.neurobiolaging.2011.08.015

[31] Cai H , Yao H, Ibayashi S, Uchimura H, Fujishima M. Photothrombotic Middle cerebral artery occlusion in spontaneously hypertensive rats: influence of substrain, gender, and distal Middle cerebral artery patterns on infarct size. Stroke 1998;29:1982–6. 9731627 10.1161/01.str.29.9.1982

[32] Inagaki T , Etgen AM. Neuroprotective action of acute estrogens: animal models of brain ischemia and clinical implications. Steroids 2013;78:597–606. 23385013 10.1016/j.steroids.2012.12.015 PMC3733348

[33] Liu R , Yang SH. Window of opportunity: estrogen as a treatment for ischemic stroke. Brain Res 2013;1514:83–90. 23340160 10.1016/j.brainres.2013.01.023 PMC3664650

[34] Lu P , Zhang CC, Zhang XM, Li HG, Luo AL, Tian YK, Xu H. Down-regulation of NOX4 by betulinic acid protects against cerebral ischemia-reperfusion in mice. J Huazhong Univ Sci Technolog Med Sci 2017;37:744–9. 29058289 10.1007/s11596-017-1798-5

[35] Yao L , Kan EM, Lu J, Hao A, Dheen ST, Kaur C, Ling EA. Toll-like receptor 4 mediates microglial activation and production of inflammatory mediators in neonatal rat brain following hypoxia: role of TLR4 in hypoxic microglia. J Neuroinflammation 2013;10:23. 23388509 10.1186/1742-2094-10-23 PMC3575244

[36] Caso JR , Pradillo JM, Hurtado O, Lorenzo P, Moro MA, Lizasoain I. Toll-like receptor 4 is involved in brain damage and inflammation after experimental stroke. Circulation 2007;115:1599–608. 17372179 10.1161/CIRCULATIONAHA.106.603431

[37] Caso JR , Pradillo JM, Hurtado O, Leza JC, Moro MA, Lizasoain I. Toll-like receptor 4 is involved in subacute stress-induced neuroinflammation and in the worsening of experimental stroke. Stroke 2008;39:1314–20. 18309167 10.1161/STROKEAHA.107.498212

[38] Ye Y , Jin T, Zhang X, Zeng Z, Ye B, Wang J, Zhong Y, Xiong X, Gu L. Meisoindigo protects against focal cerebral ischemia-reperfusion injury by inhibiting NLRP3 inflammasome activation and regulating microglia/macrophage polarization via TLR4/NF-κB signaling pathway. Front Cell Neurosci 2019;13:553. 31920554 10.3389/fncel.2019.00553 PMC6930809

[39] Azam S , Jakaria M, Kim IS, Kim J, Haque ME, Choi DK. Regulation of toll-like receptor (TLR) signaling pathway by polyphenols in the treatment of age-linked neurodegenerative diseases: focus on TLR4 signaling. Front Immunol 2019;10:1000. 31134076 10.3389/fimmu.2019.01000 PMC6522942

[40] Zhang L , Liu Y, Wang S, Long L, Zang Q, Ma J, Yu L, Jia G. Vagus nerve stimulation mediates microglia M1/2 polarization via inhibition of TLR4 pathway after ischemic stroke. Biochem Biophys Res Commun 2021;577:71–9. 34507068 10.1016/j.bbrc.2021.09.004

[41] Xu J , Liu J, Mi Y, Zhao T, Mu D, Meng Q, Wang F, Li N, Hou Y. Triad3A-dependent TLR4 ubiquitination and degradation contributes to the anti-inflammatory effects of pterostilbene on vascular dementia. J Agric Food Chem 2022;70:5896–910. 35532888 10.1021/acs.jafc.2c01219

[42] Wang P , Shao BZ, Deng Z, Chen S, Yue Z, Miao CY. Autophagy in ischemic stroke. Prog Neurobiol 2018;163-164:98–117. 29331396 10.1016/j.pneurobio.2018.01.001

[43] Matei N , Camara J, McBride D, Camara R, Xu N, Tang J, Zhang JH. Intranasal wnt3a attenuates neuronal apoptosis through Frz1/PIWIL1a/FOXM1 pathway in MCAO rats. J Neurosci 2018;38:6787–801. 29954850 10.1523/JNEUROSCI.2352-17.2018 PMC6067074

[44] Zhang K , Tu M, Gao W, Cai X, Song F, Chen Z, Zhang Q, Wang J, Jin C, Shi J, Yang X, Zhu Y, Gu W, Hu B, Zheng Y, Zhang H, Tian M. Hollow Prussian blue nanozymes drive neuroprotection against ischemic stroke via attenuating oxidative stress, counteracting inflammation, and suppressing cell apoptosis. Nano Lett 2019;19:2812–23. 30908916 10.1021/acs.nanolett.8b04729

[45] Zhang DM , Zhang T, Wang MM, Wang XX, Qin YY, Wu J, Han R, Sheng R, Wang Y, Chen Z, Han F, Ding Y, Li M, Qin ZH. TIGAR alleviates ischemia/reperfusion-induced autophagy and ischemic brain injury. Free Radic Biol Med 2019;137:13–23. 30978385 10.1016/j.freeradbiomed.2019.04.002

[46] Liu H , Wu X, Luo J, Wang X, Guo H, Feng D, Zhao L, Bai H, Song M, Liu X, Guo W, Li X, Yue L, Wang B, Qu Y. Pterostilbene attenuates astrocytic inflammation and neuronal oxidative injury after ischemia-reperfusion by inhibiting NF-κB phosphorylation. Front Immunol 2019;10:2408. 31681297 10.3389/fimmu.2019.02408 PMC6811521

