# Supplementary material for: Polydopamine-modified black phosphorus nanosheet drug delivery system for the treatment of ischemic stroke
Source: Regen Biomater. 2024 May 2;11:rbae046. doi: 10.1093/rb/rbae046 (PMC11105953; doi:10.1093/rb/rbae046)
Supplement: rbae046_Supplementary_Data [file rbae046_supplementary_data.docx]

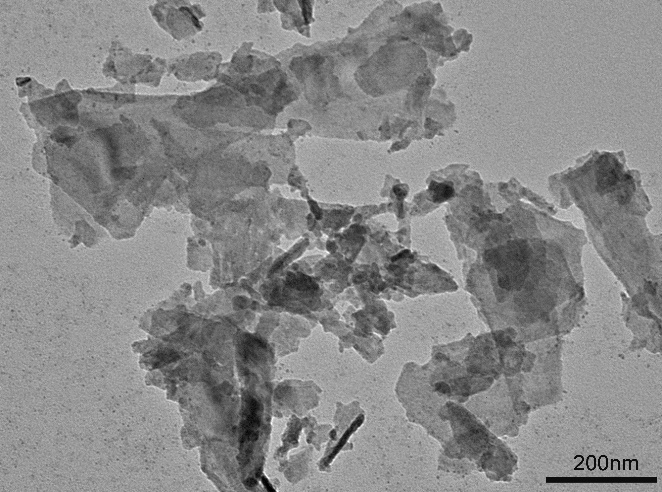
**Supplementary Material**

Fig S1 TEM images after the conditioned degradation of BP-Pte@PDA in a pH=5 solution irradiated by NIR for 48 hours. Scale bar: 200nm


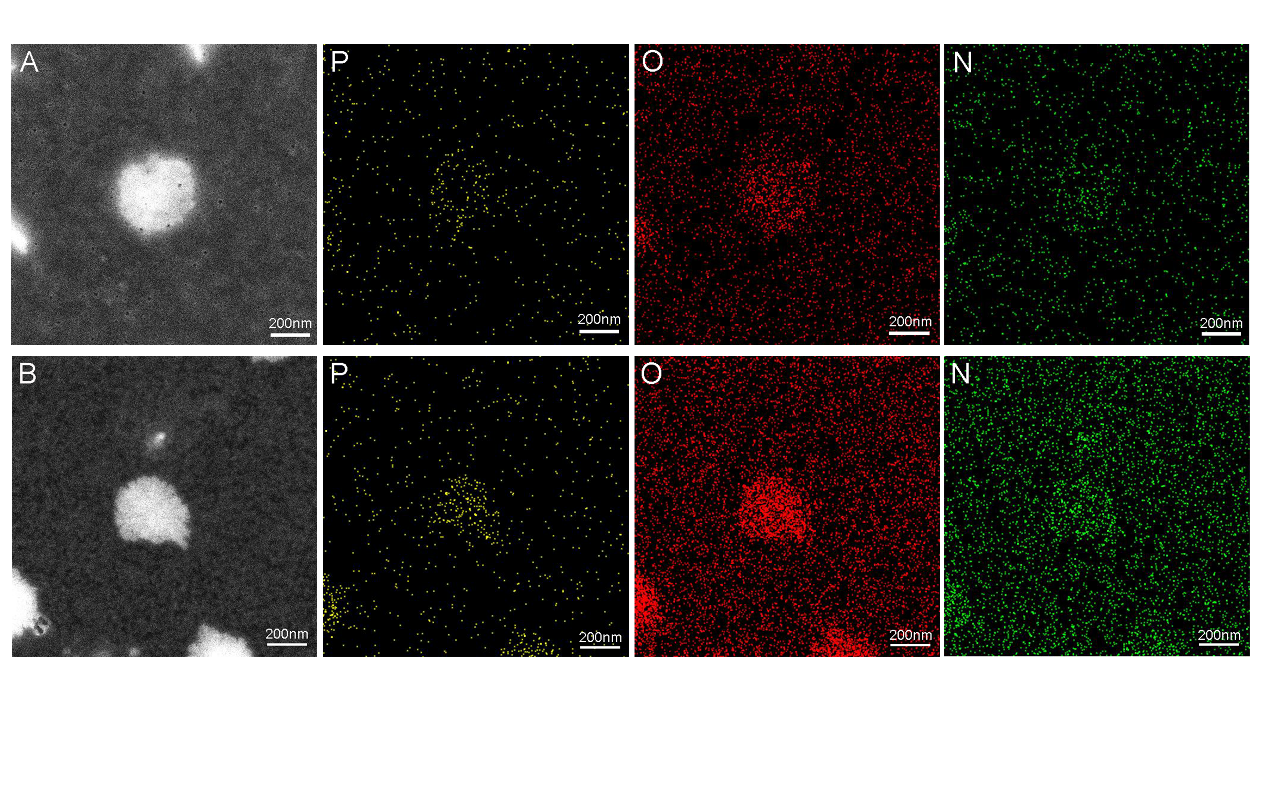
Fig S2 The high-resolution scanning TEM image and elemental mapping of BP (A) and BP-Pte (B). Scale bar: 200nm.


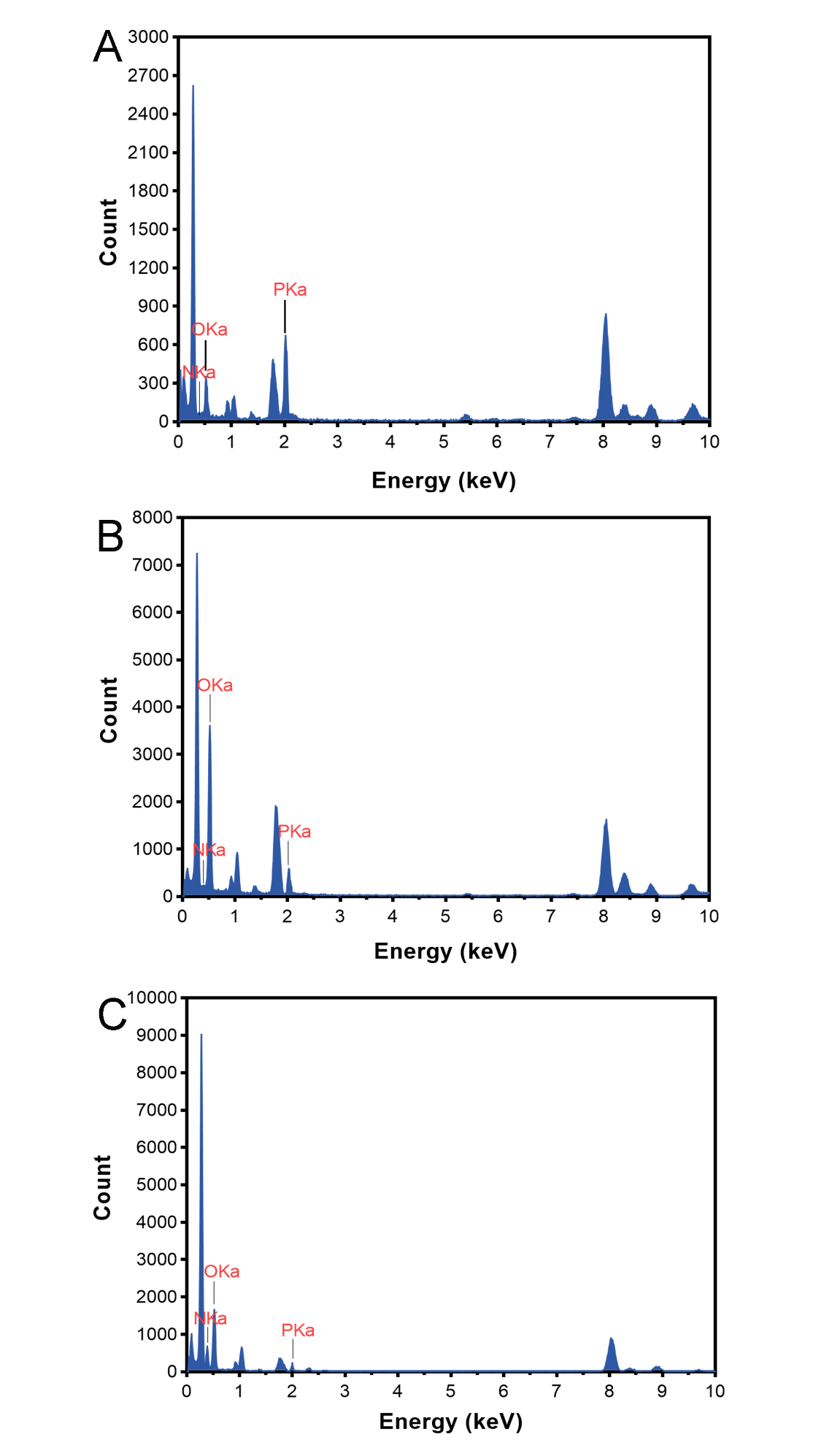
Fig S3 Mapping map (A) BP; (B) BP-Pte; (C)BP-Pte@PDA.


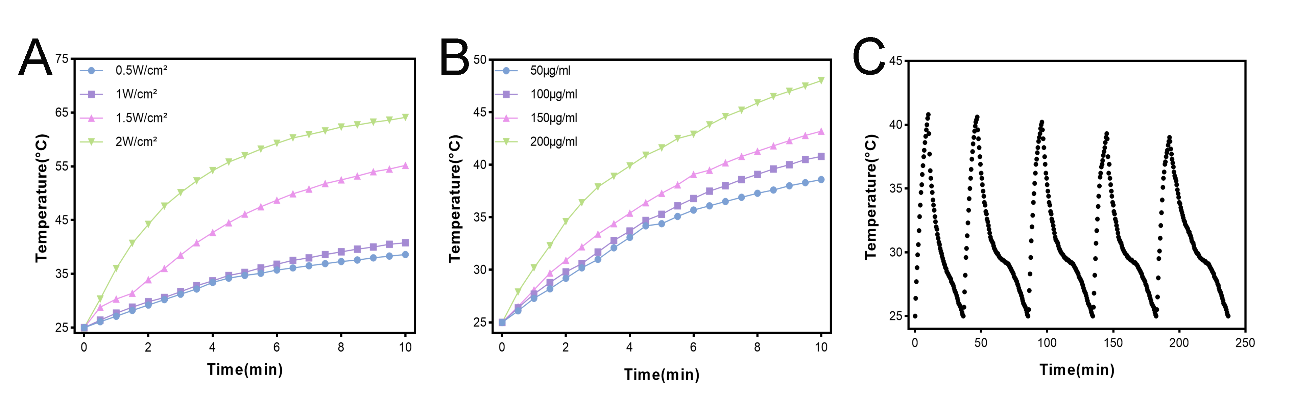
Fig S4 The photothermal properties of BP nanosheets. (A) 100 μg/mL BP nanosheets’s temperature change under NIR irradiation with different powers. (B) Temperature change curves of different concentrations of BP nanosheets under NIR (1W/cm^2^) irradiation. (C) Five cycles of heating and cooling of BP nanosheets.


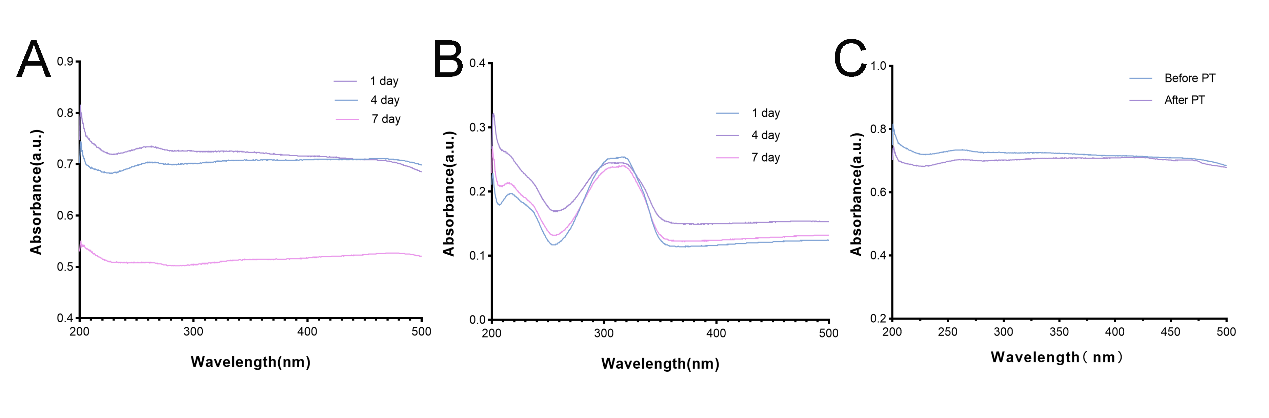
Fig S5 Stability evaluation. The wavelength change curve of different solutions in 7 days (A) BP nanosheets; (B) BP-Pte. (C) The wavelength change of BP nanosheets before and after NIR (808nm, 1W/cm^2^) irradiation.

Tab S1 In vitro drug release rate of BP-Pte@PDA drug delivery system under different pH and with or without NIR irradiation. (*n* = 3, Mean±SD.)

| Group | 7 | 6 | 5 |
| --- | --- | --- | --- |
| +NIR | 15.893±0.010 | 25.806±0.160*** | 45.952±0.314*** |
| -NIR | 21.455±0.021 | 25.735 ± 0.043**** | 38.061 ± 0.035**** |

Note: ****p* < 0.001, *****p* <0.0001, significant difference compared to pH=7 group.

| Parameters | Pte | BP-Pte@PDA+NIR |
| --- | --- | --- |
| T_1/2_ (h) | 7.965±0.547 | 2.527±0.785*** |
| C_max_(μg/mL) | 21.155±1.427 | 3.434±0.323**** |
| AUC_0-t_(μg·h/mL） | 26.777±2.481 | 11.155±0.444*** |
| MRT_0-t_(h) | 4.676±0.056 | 3.721±0.105*** |

Tab S2 Blood pharmacokinetic parameters of Pte and BP-Pte@PDA+NIR (*n* = 3, Mean±SD.)

Note: **p* < 0.05, ****p* < 0.001, *****p* <0.0001, significant difference compared to Pte group.

Tab S3 Pharmacokinetic parameters of brain tissue of Pte and BP-Pte@PDA+NIR (*n* = 3, Mean±SD.)

| Parameters | Pte | BP-Pte@PDA |
| --- | --- | --- |
| T_1/2_ (h) | 3.348±0.387 | 2.445±0.325* |
| C_max_(μg/mL) | 0.077±0.008 | 5.701±0.844*** |
| AUC_0-t_(μg·h/mL） | 0.558±0.039 | 42.683±2.668**** |
| MRT_0-t_(h) | 8.338±0.069 | 8.235±0.151 |

Note: ****p* < 0.001, *****p* <0.0001, significant difference compared to Pte group.


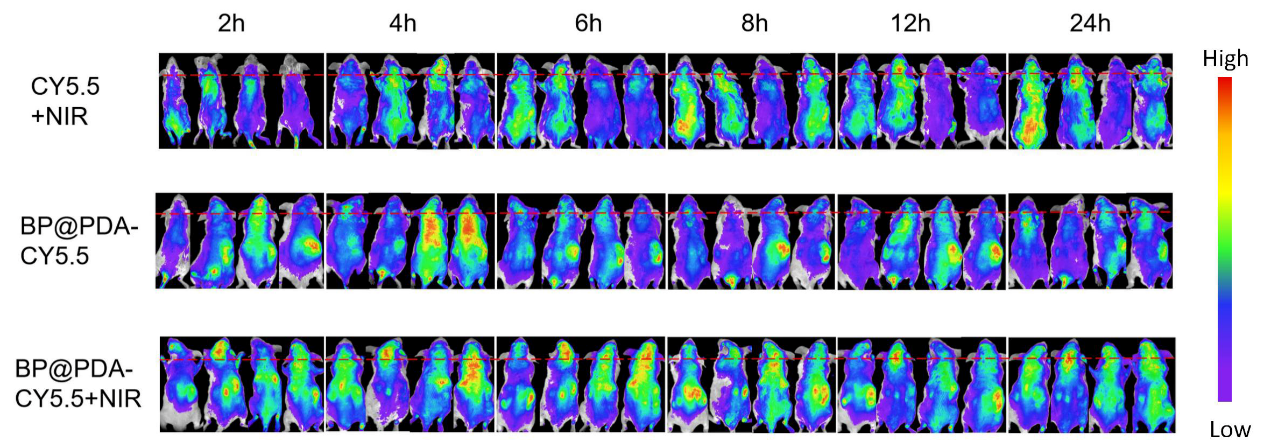
Fig S6 In vivo whole body fluorescence imaging of mice from 2 to 24 h (n=4).


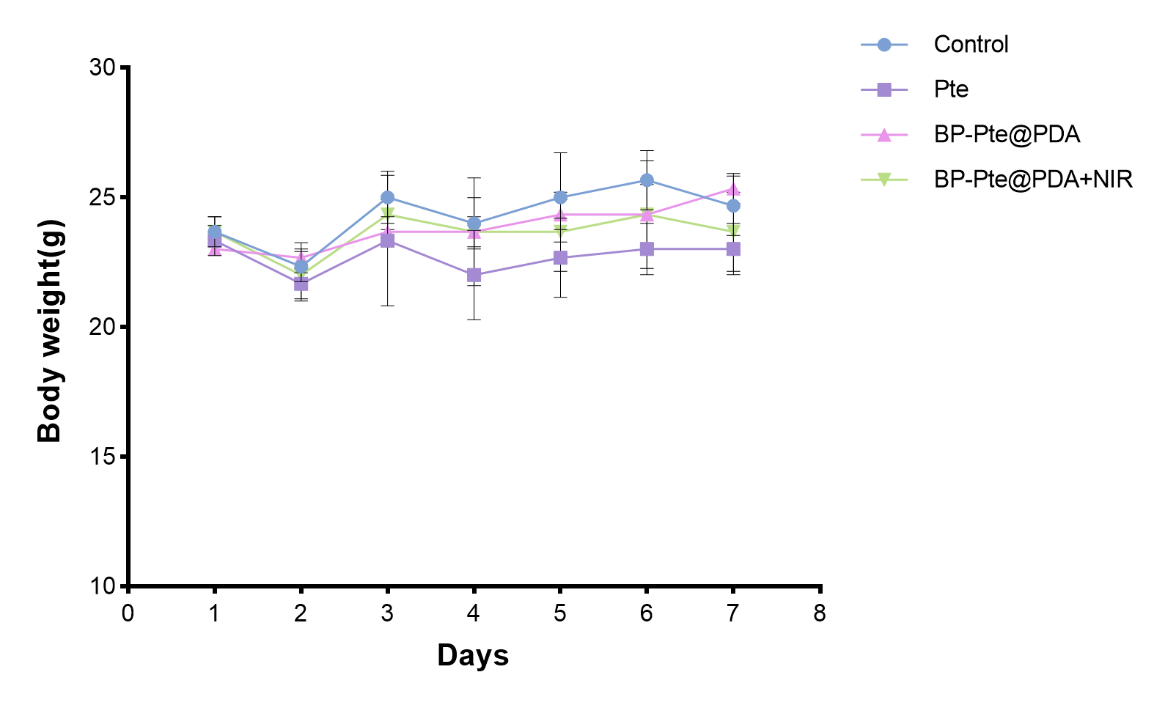
Fig S7 The weight change of mice after 7 days of administration (n=3).
